# Exploring *TTN* variants as genetic insights into cardiomyopathy pathogenesis and potential emerging clues to molecular mechanisms in cardiomyopathies

**DOI:** 10.1038/s41598-024-56154-7

**Published:** 2024-03-04

**Authors:** Amir Ghaffari Jolfayi, Erfan Kohansal, Serwa Ghasemi, Niloofar Naderi, Mahshid Hesami, MohammadHossein MozafaryBazargany, Maryam Hosseini Moghadam, Amir Farjam Fazelifar, Majid Maleki, Samira Kalayinia

**Affiliations:** 1 Rajaie Cardiovascular Medical and Research Center, Iran University of Medical Sciences, Tehran, Iran; 2 Cardiogenetic Research Center, Rajaie Cardiovascular Medical and Research Center, Iran University of Medical Sciences, Tehran, Iran

**Keywords:** *TTN*, Titin, Cardiomyopathy, Variant, Genetic, Genetics, Cardiology, Medical research, Molecular medicine

## Abstract

The giant protein titin (TTN) is a sarcomeric protein that forms the myofibrillar backbone for the components of the contractile machinery which plays a crucial role in muscle disorders and cardiomyopathies. Diagnosing *TTN* pathogenic variants has important implications for patient management and genetic counseling. Genetic testing for *TTN* variants can help identify individuals at risk for developing cardiomyopathies, allowing for early intervention and personalized treatment strategies. Furthermore, identifying *TTN* variants can inform prognosis and guide therapeutic decisions. Deciphering the intricate genotype–phenotype correlations between *TTN* variants and their pathologic traits in cardiomyopathies is imperative for gene-based diagnosis, risk assessment, and personalized clinical management. With the increasing use of next-generation sequencing (NGS), a high number of variants in the *TTN* gene have been detected in patients with cardiomyopathies. However, not all *TTN* variants detected in cardiomyopathy cohorts can be assumed to be disease-causing. The interpretation of *TTN* variants remains challenging due to high background population variation. This narrative review aimed to comprehensively summarize current evidence on *TTN* variants identified in published cardiomyopathy studies and determine which specific variants are likely pathogenic contributors to cardiomyopathy development.

## Introduction

Cardiomyopathies refer to a diverse range of complex diseases affecting heart muscle, which can lead to abnormalities in the structure and function of the myocardium. These abnormalities occur in the absence of other conditions like coronary artery disease, hypertension, or valvular heart disease^1,2^. The American Heart Association (AHA) has categorized cardiomyopathies into genetic, acquired or mixed forms like virally induced post-myocarditis cardiomyopathy. The European Society of Cardiology Organization (ESCO) proposed an alternative classification system dividing cardiomyopathies into two subgroups—familial/genetic cardiomyopathies and non-familial/non-genetic cardiomyopathies^3,4^. Based on morpho-functional phenotypes^5^, cardiomyopathies are classified into hypertrophic cardiomyopathy (HCM), dilated cardiomyopathy (DCM), restrictive cardiomyopathy (RCM), and arrhythmogenic right ventricular (ARVC) which each one has their specific features^6^. The hallmark features of cardiomyopathies are genetic and clinical heterogeneity, variable expressivity, and incomplete penetrance. Numerous genes and mutations have been identified that can cause the various types of cardiomyopathies. The majority of known mutations are linked to DCM and HCM, while fewer are associated with RCM and ARVC. Cardiomyopathies demonstrate considerable genetic heterogeneity—mutations in various different genes can lead to cardiomyopathy. There is also phenotypic heterogeneity, where mutations in the same gene can result in diverse types and degrees of severity of cardiomyopathy^7^. Cardiomyopathy following myocarditis is probably the result of an interaction interplay between the viral infection and a person's inherent susceptibility. Certain subgroups induced by viral infection may be influenced, at least partially, by genetic factors, suggesting that the elimination of the virus and the immune response could be genetically predetermined^8^.

Among the genes involved in cardiomyopathies, the *TTN* gene plays a central role which is attributable to its key structural properties and mechanical function within the striated muscle sarcomeres^9^. *TTN* is a major human muscle disease-related gene that encodes the largest human protein, Titin, which is a fundamental structural and functional unit of striated muscles^10,11^. Due to the size and complexity of this gene, its sequencing was difficult to study the mutations and variants. The initial family studies were performed with primer pairs searching on the exons contained in a 280 kb genomics 2q31 region. This indeed led to the identification of titin mutations causing DCM by Gramlich et al.^12^ Subsequently, the introduction of NGS has allowed for the exploration of larger cohorts and various clinical entities.

Following the development of next-generation sequencing (NGS), as a potent tool for sequencing large and complex genes, *TTN* gene sequencing which was previously impossible to conduct a comprehensive analysis, has been performed. This improvement in study tools has led to identifying more than 60,000 *TTN* missense variants reported in the 1000 Genomes Project^13,14^. Determining which TTN variants actually cause disease versus which are benign is challenging. The goal of this review is to discuss the current state of understanding regarding the challenges in establishing clear associations between particular TTN mutations and specific cardiomyopathy subtypes in a clinical context.

## Method and materials

### Systematic search, selection criteria and data collection

The study systematically collected *TTN* variants associated with cardiomyopathy from the Human Gene Mutation Database (HGMD) and public archive of interpretations of clinically relevant variants (ClinVar). In prioritizing data reliability, only variants with documented reference articles were included, while those lacking reference articles were excluded. The search strategy, extending until February 2023, employed key parameters such as Position on Chromosome, Human Genome Variation Society (HGVS) DNA, HGVS Protein, exon or intron number, and dbSNP identifiers.

### Variant annotation and pathogenicity assessment

The annotation of *TTN* variants involved a comprehensive pathogenicity assessment using multiple tools. This included the application of the American College of Medical Genetics and Genomics (ACMG) guidelines, consultation of ClinVar for variant interpretation, insights from Mutation Taster regarding potential pathogenicity, the use of the Combined Annotation Dependent Depletion (CADD) scoring system for deleteriousness prediction, and evaluation through Genomic Evolutionary Rate Profiling (GERP) to assess evolutionary conservation which are explain more in the following.

We determineded the ACMG score for each variant using franklin, an online database (https://franklin.genoox.com/clinical-db). After adding the name in this website, varints ACMG score anongside with other features are provided.

#### ACMG score

The American College of Medical Genetics and Genomics (ACMG) previously established guidelines for interpreting sequence variants. With the rapid advancements in sequencing technology over the past decade, this report suggests the adoption of standardized terms such as “pathogenic,” “likely pathogenic,” “uncertain significance,” “likely benign,” and “benign” to characterize variants found in genes associated with Mendelian disorders. Additionally, the recommendation outlines a systematic approach for classifying variants into these categories, relying on various types of evidence, including population data (Population, disease-specific, and sequence databases), computational data (using silico tools for missense prediction, splice site prediction and nucleotide conservation prediction), functional data, and segregation data^15,16^.

In this classification a variant is considered pathogenic if it meets the requirement of having a very strong criterion (PVS1) along with at least one strong criterion (PS1-PS4), or alternatively, two or more moderate criteria (PM1-PM6), or a combination of one moderate criterion and one supporting criterion (PP1-PP5). Another condition is that a variant can be classified as pathogenic if it satisfies the condition of having at least two strong criteria (PS1-PS4). Additionally, a variant can be considered pathogenic if it meets the criteria of having one strong criterion (PS1-PS4) and either three moderate criteria (PM1-PM6), two moderate criteria and at least two supporting criteria (PP1-PP5), or one moderate criterion and at least four supporting criteria (PP1-PP5)^16^.

A variant is considered likely pathogenic if it satisfies the condition of having one very strong criterion (PVS1) in combination with one moderate criterion (PM1-PM6). Alternatively, a likely pathogenic variant may exhibit one strong criterion (PS1-PS4) along with one to two moderate criteria (PM1-PM6). Another criterion designates a variant as likely pathogenic if it possesses one strong criterion (PS1-PS4) and at least two supporting criteria (PP1-PP5). Furthermore, likely pathogenic variants may be identified if they meet the requirement of having three or more moderate criteria (PM1-PM6). Additionally, a variant is classified as likely pathogenic if it has two moderate criteria (PM1-PM6) and at least two supporting criteria (PP1-PP5), or if it exhibits one moderate criterion (PM1-PM6) along with at least four supporting criteria (PP1-PP5)^16^. More information is provided in *“Standards and guidelines for the interpretation of sequence variants: a joint consensus recommendation of the American College of Medical Genetics and Genomics and the Association for Molecular Pathology”*^16^.

The ACMG score for each variant is determined using Franklin, an online database available at https://franklin.genoox.com/clinical-db. Upon entering the variant's name on this website, the ACMG score, along with other relevant features, is provided.

#### CADD score

CADD, or Combined Annotation Dependent Depletion, serves as a tool for evaluating the deleteriousness of various genetic variants, including single nucleotide changes, multi-nucleotide substitutions, and insertion/deletion variants within the human genome. In contrast to many other annotation tools that often rely on a singular type of information or have limited applicability, CADD offers a versatile metric that objectively combines diverse annotations. The framework integrates multiple annotations into a unified metric by comparing variants that have undergone natural selection with simulated mutations. It incorporates information from more than 60 genomic features to assess single nucleotide variants and short insertions and deletions across the reference assembly. The C-scores generated by CADD demonstrate robust correlations with allelic diversity, pathogenicity of coding and non-coding variants, and experimentally measured regulatory effects. Notably, C-scores of variants associated with complex traits in genome-wide association studies (GWAS) are significantly higher than matched controls, showing correlation with study sample size, indicative of improved accuracy in larger GWAS. CADD employs a machine learning model that distinguishes between simulated de novo variants, potentially encompassing neutral or harmful alleles, and variants persisting in human populations since the split from chimpanzees.

CADD's capability to quantitatively prioritize functional, deleterious, and disease-causing variants spans a wide range of functional categories, effect sizes, and genetic architectures. This tool enhances the scoring of coding variants through features derived from the ESM-1v protein language model and improves the scoring of regulatory variants using features from a convolutional neural network trained on open chromatin regions. For more information CADD has been detailed in four publications^17–20^.

#### MutationTaster

MutationTaster is a web-based application designed to assess the disease-causing potential of DNA sequence variants. It employs in silico tests to estimate the impact of a variant on the gene product or protein, conducting assessments at both the protein and DNA levels. Unlike tools limited to single amino acid substitutions, MutationTaster can handle a variety of variants, including synonymous and intronic ones^21^. The software, written in Perl programming language and utilizes integrated databases (Ensembl, UniProt, ClinVar, ExAC, 1000 Genomes Project, phyloP and phastCons) to filter out known harmless polymorphisms. Various tests, such as amino acid substitution, conservation, domain functionality, splicing effects, and regulatory element abrogation, are performed on the remaining single-nucleotide polymorphisms (SNPs). The results are evaluated by a Naive Bayes classifier, and the output indicates whether the alteration is known or predicted to be harmless or disease-causing, providing detailed information about the mutation. While the tool demonstrates a raw accuracy of approximately 90%, considering knowledge about common polymorphisms and known disease mutations significantly improves the rate of correct classifications. However, it is important to note that predictions of clinical effects suffer from a lack of specificity, a common constraint across various prediction methods^22,23^.

#### GERP

Comparative genomic approaches have historically identified mutation sites under purifying selection by examining conserved sequences across distantly related species. Additionally, the performance of such approaches may be limited for short-lived functional elements that don't exhibit sequence conservation across numerous species. Genomic Evolutionary Rate Profiling (GERP) score is associated with the strength of selection (Nes). Results indicate that the GERP score is linked to the intensity of purifying selection. Nevertheless, variations in selection coefficients or turnover of functional elements over time can significantly impact the GERP distribution, leading to unexpected relationships between GERP and Nes^24^. The GERP score is characterized as the decrease in the count of substitutions in the multi-species sequence alignment in comparison to the neutral expectation. GERP^++^ scores span from − 12.3 to 6.17, with elevated scores signifying a greater level of evolutionary constraint.

### Data integration

Data integration encompassed the consolidation of relevant information, including Position on Chromosome, HGVS DNA, HGVS Protein, exon or intron number, and dbSNP identifiers. Rigorous quality control measures were then applied to ensure the accuracy and consistency of data extraction and annotation.

### Statistical analysis

Descriptive statistics were employed for a comprehensive analysis, summarizing the distribution of TTN variants in terms of positions, types, and associated pathogenicity.

### Ethical considerations

Ethical considerations are considered in the study, with a commitment to adhering to Data reliability and responsible data handling. In the present study, it is important to note that no human subjects were involved, as this investigation is a comprehensive review rather than an experimental study. The research focused on analyzing reported variants available on PubMed, and ethical approval or consent from human participants was not applicable.

## Results

### The molecular structure of titin

The *TTN* gene located on the second human chromosome in the 2q31 area. This gene contains 364 exons, which their translation produces a 4200-kDa protein with ~ 38,000 amino acid residues, the largest polypeptide found in the human body. The Titin giant protein, also known as connectin, is the third most abundant protein found in striated muscle among the vertebrates, after myosin and actin. The Titin is a flexible filament that is more than 1 µm long and 3–4 nm wide and spans half of the sarcomere as the repeating contractile unit that gives striated muscle characteristic striped appearance^25^.

Titin has a complex multidomain structure which is composed of four main structural and functional regions: the N-terminal Z-line acts as an anchor for the sarcomeric Z-disk; the I-band provides elastic properties; the A-band stabilizes the thick filament; and the C-terminal M-line extremity overlaps in an antiparallel orientation with another titin molecule's C-terminus, allowing for modulation of titin expression and turnover via the tyrosine kinase domain^26^.

The N-terminus contains immunoglobulin (Ig) domains, fibronectin (FN) domains, and a Z-disk region^27^. The rest of the titin molecule includes an elastic I-band region, a spring-like Pro-Glu-Val-Lys (PEVK) domain, three unique sequences called Novex 1, 2, and 3, cardiac-specific N2B and N2A domains, a thick A-band region, and an M-band region where the C-terminus is embedded.

Extensive alternative splicing in the 364 exons of *TTN* leads to forming various molecular isoforms. Previous studies have shown three main titin isoforms expressed in cardiomyocytes: the adult N2B isoform, the adult N2BA isoform, and the fetal cardiac titin (FCT) isoform. The distinct characteristics of each titin isoform arise from differences in their I-band sequences, while the Z-disk, A-band, and M-line regions are highly conserved across all isoforms^28^. Due to the longer extensible I-band region, the N2BA isoform is more compliant than N2B. The N2BA isoform contains additional spring-like elements in the PEVK and tandem Ig regions, leading to lower passive tension in cardiomyocytes compared to other isoforms^29–31^.

**Figure 1:**
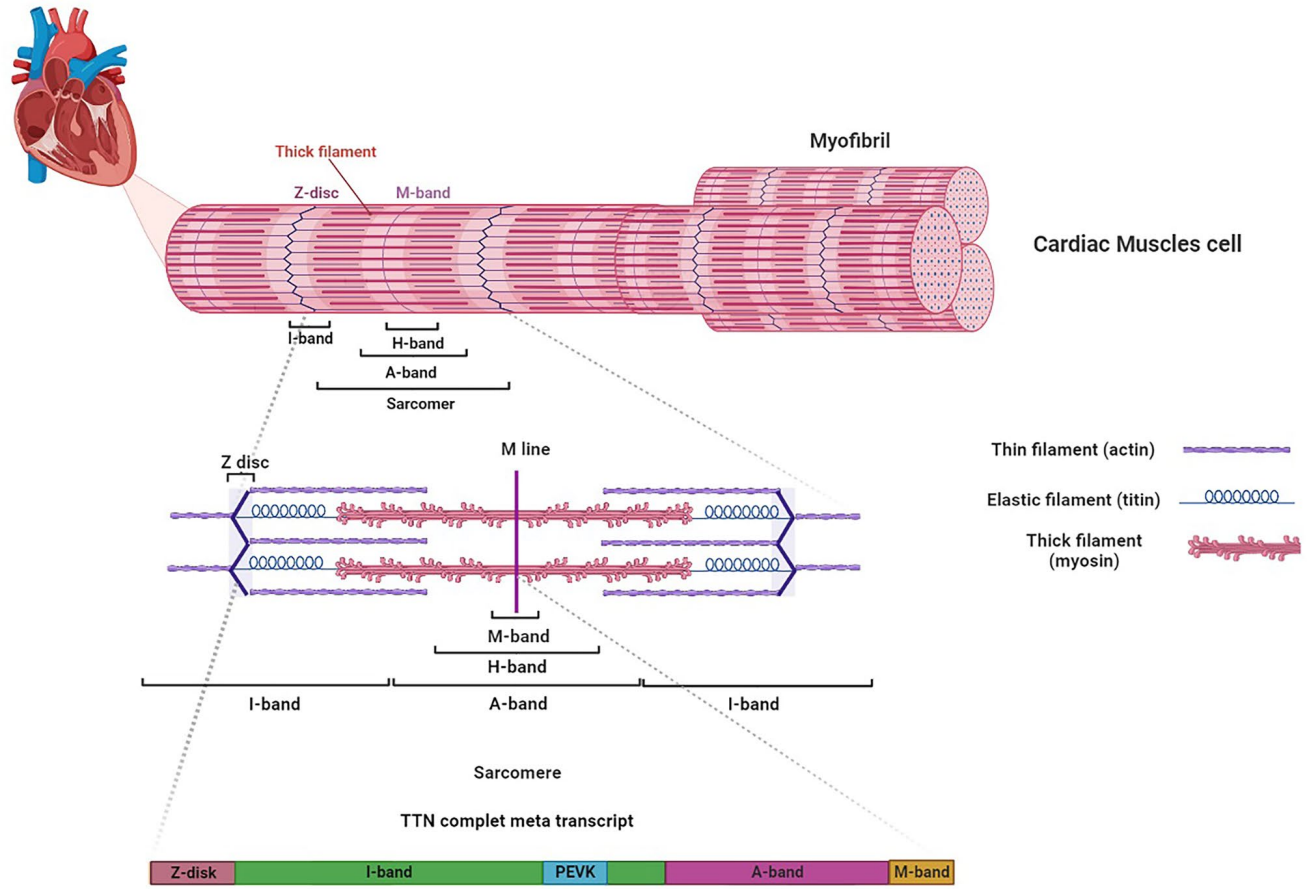
Molecular structure of sarcomere and the interaction of Titin with thin and thick filaments.

Molecular structure of sarcomere and the interaction of Titin with thin and thick filaments is demonstrated in Fig. 1.

#### Z-disk

The Z-disk region spans 826 amino acids horizontally across the structure and contains seven Ig domains separated by Z-insertion sequences. As the site of numerous structural and functional interactions with myofibrillar and sarcolemmal proteins, the Z-disk is critical for myofibril assembly, stability, and signaling. Z-disks anchor essential proteins like titin-Tcap (telethonin), which enables key Z-disk functions including mechanosensing. Mechanosensing involves recruiting other interacting and signaling partners to the Z-disk in response to mechanical stimuli. Overall, Z-disks play indispensable roles in anchoring titin and enabling vital structural and sensory functions^32–34^.

The Z-disk interacts with small ankyrin proteins, spectrin, desmin, and obscurin, connecting it to other cytoskeletal structures. Filamin C links the Z-disk to costameres via integrins and sarcoglycans, participating in mechanosensory pathways. Additionally, the Z-disk binds nebulin, which helps stabilize Z-disk anchorage through interactions with actin, desmin, CapZ and myopalladin. α-Actinin binding also enhances Z-disk mechanical stability. Overall, the Z-disk forms critical protein interactions that provide structural support and sensory functions^35–40^.

#### I-band

The I-band region of titin displays extensive alternative splicing, generating diverse isoforms that confer tissue-specific mechanical properties in cardiac and skeletal muscles. Through alternative splicing mechanisms, a spectrum of isoforms emerges, tailoring titin's mechanical functions to meet the needs of different muscle types. The I-band thus acts as a central adapter, converting titin into specialized molecular springs via splicing variability. This interactive segment contains a meta-transcript with principal cardiac and skeletal isoforms. Key components include immunoglobulin folds, the cardiac N2B zone, and the skeletal N2A zone containing nonrepetitive sequences and immunoglobulin domains. The proline-glutamate-valine-lysine (PEVK) domain follows, acting as a spring-like element. Together, the I-band components enable the elasticity of titin^38,41^.

The I-band region has distinct proximal and distal segments with specialized roles. The proximal I-band maintains sarcomere integrity, while the medial/distal I-band acts as a bidirectional molecular ruler setting resting length and passive tension^42^. The I-band also functions as a biochemical stress sensor through interactions with αβ-crystallin, a chaperone that stabilizes I-band immunoglobulin domains. Additionally, metabolic enzymes like DRAL, FHL1, and FHL2 associate with I-band sarcomere regions via the Gαq-MAPK pathway^37,43,44^. Indeed, though I-band interactions with the Ca^+2^-dependent proteases Calpain-1 and Calpain-3, I-band not only contributes to a sarcomeric quality control pathway but also serves as a reservoir for inactive forms of Calpain-3^45,46^.

#### A-band

The A-band spans the sarcomere from M-line to M-line, containing thick filaments of myosin. Within the A-band, titin forms a network that maintains the structural integrity of the thick filaments and regulates their length. The A-band exclusively contains fibronectin type III (FnIII) motifs. Immunoglobulin (Ig) and FnIII motifs are arranged in two super-repeats bisected by Ig folds. Unlike the elastic I-band, the A-band is inextensible, providing myosin binding sites that function as stable anchors. A-band super-repeat domains interact with and position sarcomeric myosin binding protein C (MyBP-C). The A-band also contains binding sites for muscle ring finger proteins MURF1 and MURF2. MURF1 likely facilitates quality control and protein turnover at the sarcomere center, while MURF2 interactions aid formation of mature A-band structures^36,38^.

#### M-band

The M-band integrates structural, signaling, metabolic and protein quality control functions. It contains a putative serine/threonine kinase domain and immunoglobulin cross-hatched rectangle (CII) domains interspersed with M-insertion sequences^47^. While its kinase activity is debated, the M-band kinase domain likely participates in stress sensing through Ca^2+^-calmodulin-regulated mechanochemical signaling^38,48^. During sarcomerogenesis, myomesin constructs an M-band scaffold linking titin to myosin thick filaments, establishing the myomesin-titin-myosin stability axis^49^. The M-band also senses metabolic stress via ligands DRAL/FHL2 that tether metabolic enzymes, and enables ubiquitin-mediated turnover through interactions with nbr1, p62, MURF1 and MURF2^50^. MURF2 binding facilitates M-band's role in cardiac development^51^. Additionally, the extreme C-terminal TTN/calpain-3/p94 interaction participates in M-band-associated protein turnover^37,52^.

### The molecular function of titin

Since the discovery of titin, the complexity and diverse functional roles of titin in health and disease continue to emerge. As the third filament system of the sarcomere alongside actin and myosin, titin forms a unique filament network in cardiomyocytes that engages in mechanical and signaling roles^10^. During muscle development, titin likely controls the assembly of actin and myosin contractile proteins, regulating sarcomere size and thick filament structure. In mature muscle, titin contributes to elasticity mechanisms affecting sarcomere resting lengths and tension-related processes^25^.

The enormity and intricate three-dimensional structure of titin provides structural support to maintain sarcomere integrity during contraction while generating passive tension during stretching. Additionally, the numerous titin-binding proteins arranged in signaling hotspots allow titin to participate in mechanosensing and signal transduction^26,53^. Thus, titin has multifaceted roles beyond viscoelastic force generation: (a) centering thick filaments for optimal active force; (b) assembling sarcomeres; (c) mechanochemical signaling through binding partners; and (d) potentially enabling length-dependent activation underlying the Frank-Starling law^54^.

### Comparative analysis of *TTN* variants

In this study we found 611 distant TTN variant which were not benign and they were pathogenic, likely pathogenic or variant of uncertain significance (VUS).

85% of the variants were reported in exon fragments, while 15% were reported in intron fragments. In ACMG classification, 69.6% of the variants were classified as Pathogenic, 21.6% as Likely Pathogenic, and 8.8% as Variants of Uncertain Significance (VUS). Substitution accounted for 57.25% of the variants, deletion for 29.62%, duplication for 7.36%, and insertion for 5.72%.

**Figure 2:**
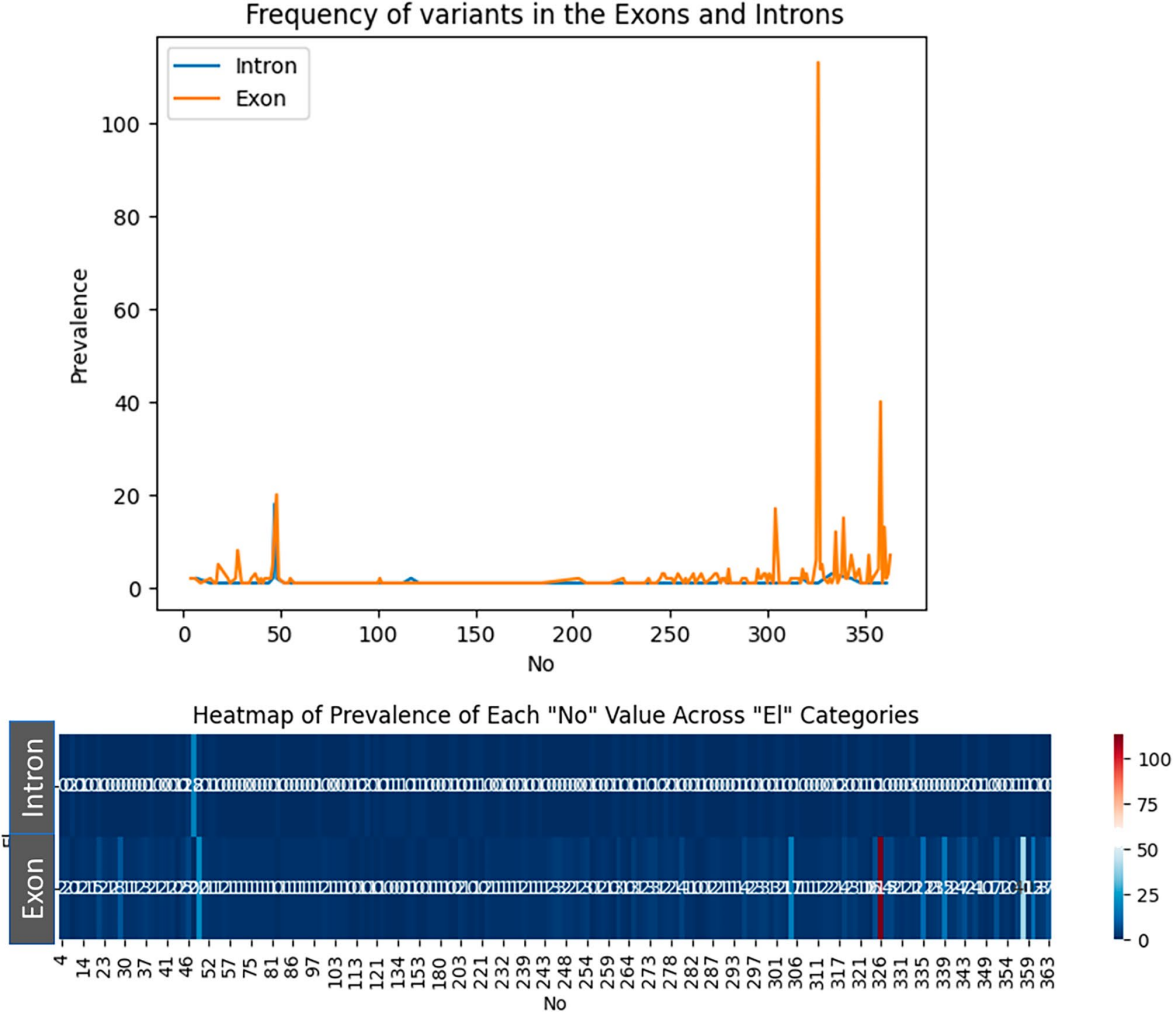
Prevalence of variants in different exons and introns in *TTN*.

The majority of variants occurred in the interval from exon 200 to the end of the molecule, with the hotspot regions identified at exon 326 and 358 being the most common points for variations (Fig. 2).

**Figure 3:**
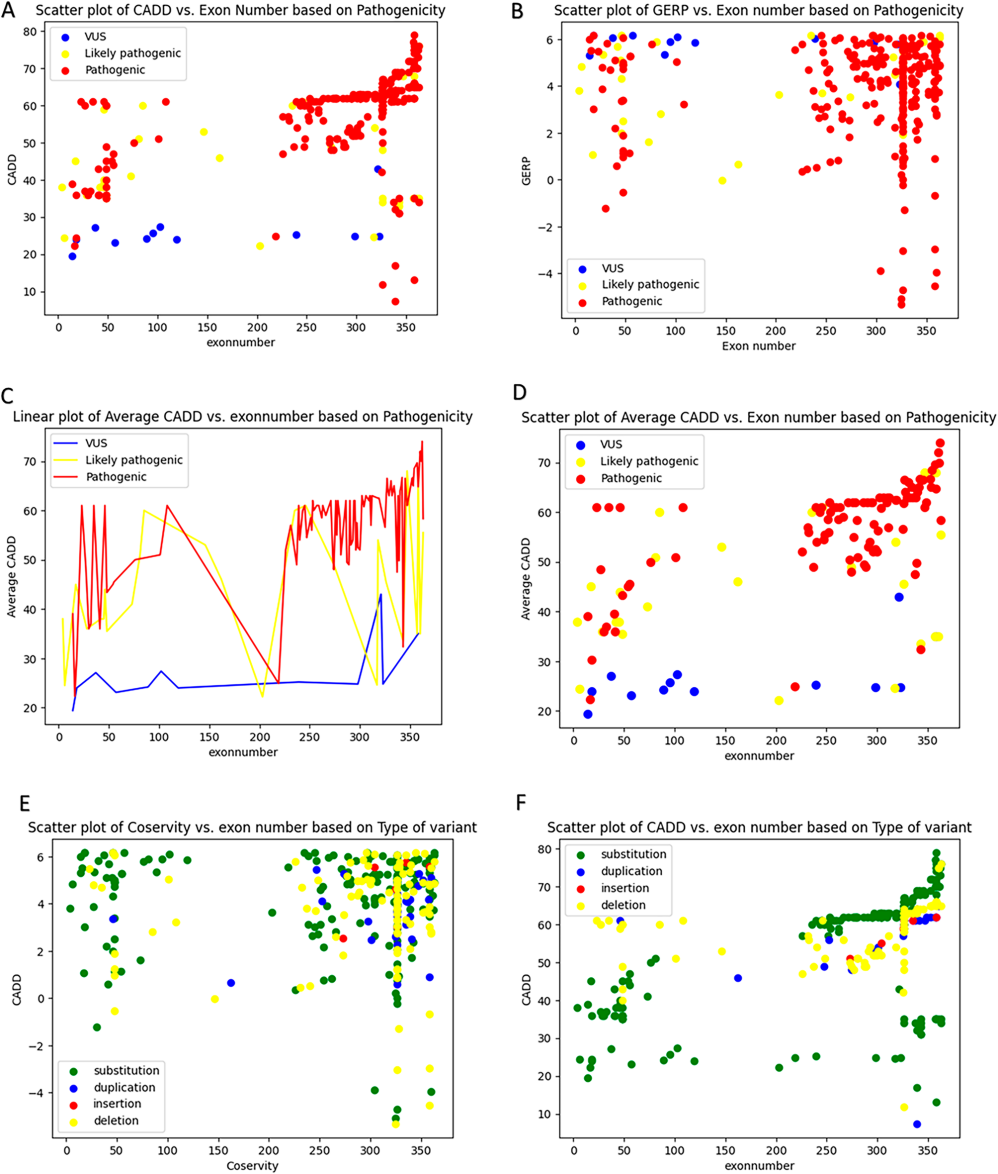
Comparative analysis of *TTN* variants with their pathogenicity, type of alternation, and conservity.

Most pathogenic variants are located after the exon 326 to the end of the molecule which has higher CADD number compared to others (Fig. 3A). The Genomic Evolutionary Rate Profiling (GERP) score is used to compare the gene nucleotides among the species in the TTN gene^24^. It is supposable that the nucleotides and exons which are conserved in the evolution, can be considered a vital element for survive and loss of function of these components are associated with death and the prevention of its inheritance. In the comparison of the conservity of the gene nucleotides, it can be concluded that most the variants have a notable GERP score which indicates their conservity (Fig. 3B).

In comparing the average CADD score of various exons, it can be concluded that exons with higher CADD scores are located in the end of the gene and the middle part of the gene, the average CADD score is not notable. The first few exons of the gene have a higher CADD score but in the last exons, the CADD score is increased considerably especially in the last 50 exons. VUS variants have less CADD score and likely pathogenic variants also have lesser scores compared to pathogenic variants (Fig. 3C,D).

In the comparing type of genetic alternation in variants, it can be concluded that the most common alternations are substitution and deletions. Most of the deletions have high score numbers while substitutions have various CADD scores. Most of the insertion and duplications also have notable CADD score because of frameshift events while in the substitutions we can observe some lesser CADD score which is not exists in other types of alternations. As demonstrated, most of the pathogenic variants in the first parts of the gene are deletions but the most pathogenic variants in the last parts of the gene have substitutions (Fig. 3E,F).

### The biogenesis pathways of *TTN*

#### Role of alternative splicing

*TTN* gene consists of 364 exons as translatable parts according to NCBI^55^ and is estimated to code 34,350 amino acid residues according to UniProt^56^. *TTN* can be spliced in different ways to produce different transcript forms. Since alternative splicing of *TTN*, the protein has various sizes. The I-band, M-line, and Z-disc areas of Titin are the most variable parts, which lead to various isoforms with a wide range of elasticity. Due to variations in the I-band area, different muscle types have varying degrees of elasticity. The Titin gene's I-band encoding region is the site of many splicing processes resulting in isoforms with various spring compositions. This process even can discriminate cardiac Titin with skeletal muscle Titin.

All cardiac Titin isoforms have exon 49, which contains the N2B sequence; however, skeletal muscle does not^57^. The cardiac isoform known as N2B Titin is a small 2970-kDa weight protein produced by splicing exons 49/50. Deletion of Titin N2B region causes diastolic dysfunction and cardiac atrophy^58^. Another isoform is N2BA which is made up of exons 102 to 109, which code for the N2A element. A specific property of this isoform is that it contains more PEVK segments and is longer with more Ig domains^58^.

#### I-band and its isoforms in cardiac compliance and DCM

Protein composition patterns can change among different populations and even in various stages of human life. The isoform transforming of sarcomeric proteins in the troponin complex, Myosine heavy chain (MHC), Myosine light chain (MLC) and Titin from fetal to adult through transcriptional changes or alternative splicing is the essential element of myofibril maturation^59^.

A study by Lahmers et al.^59^ revealed that fetal titin isoforms are expressed in neonates, containing additional spring elements in the tandem Ig and PEVK regions. This leads to lower stiffness compared to adults, explained by the unique spring composition of fetal cardiac titin in neonates. Changes in titin expression during development likely impact functional transitions and diastolic filling as the heart matures. The fetal cardiac titin isoform, with its extra Ig and PEVK spring elements, gradually disappears postnatally in a species-dependent manner.

In the human heart, the ratio of titin isoform expression is established based on passive tension. There is a high correlation between titin-based passive tension and I-band region size, with lower tension associated with a larger, more elastic I-band. In healthy adult hearts, the N2BA and N2B titin isoforms express at 30–40% and 60–70% respectively. The relative levels of these two isoforms are a key determinant of cardiomyocyte stiffness^60^. Titin plays a central role in the passive ventricular tension. Animal studies have proved that the N2BA isoform is present in the near-term fetus 6 days before birth but after birth disappears and is replaced by a smaller N2B isoform, which predominates in 1-week-old neonate and adults. Adult cardiomyocytes have 15 times more passive tension compared to fetal cardiomyocytes which is confirmed by immunofluorescence microscopy. This transformation is compatible with the heart's function in each stage of life which after birth needs more passive tension to pump the blood effectively through the vessels^61^.

Alternative splicing of the TTN gene plays significant roles in cardiac diseases like dilated cardiomyopathy (DCM). In DCM, the more compliant N2BA isoform is upregulated, decreasing passive stiffness and increasing chamber compliance. Overall, variable expression and splicing of titin isoforms critically influence myocardial passive tension and compliance^30,31,62,63^.

Hidalgo et al.^64^ conducted sophisticated experiments to identify the mechanisms influencing myocardial passive stiffness by modifying the phosphorylation state of titin. The study revealed that titin serves as a substrate not only for protein kinase A but also for protein kinase G and protein kinase C α (PKCα). The researchers pinpointed the PEVK region of titin as the primary site for PKCα phosphorylation, demonstrating that phosphorylation at this site enhances passive tension in the myocardium.

#### Novex variants and tiny titin results alternative splicing

The whole sequence of the human *TTN* gene contains three isoform-specific mutually exclusive exons named novel exons (novex), which encode for the I-band sequence. Novex1 is presented in exon 45, novex-2 is located in exon 46, and novex-3 is placed in exon 48. The novex-1 and novex-2 Titin isoforms are encoded by transcripts that either include the novex-1 or novex-2 exons. Early stop-gain codon in the novex-3 transcript produces a remarkably tiny isoform (700 kDa) known as novex-3 Titin. The 'tiny Titin' isoform, expressed in all striated muscles, stretches from the Z-disc to the novex-3 domain (C-terminus). Therefore, stress-induced sarcomeric rearrangement may be mediated by novex-3 Titin because of its regulatory involvement in calcium level and GTPase-associated myofibrillar pathways^65^. Furthermore, novexes 2 and 3 may be linked to DCM or ARVC based on the expression levels of novex variations in human cardiac tissues affected by cardiomyopathies. Previous research suggests that novex variations may be attributable to cardiomyopathy^66^.

#### Splicing regulation of alternative splicing

Encoding Titin by a single gene into various forms is the result of different mRNA splice pathways which leads to Titin isoform classes^57^. The titin gene contains 409 introns, enabling generation of 57 distinct mRNA transcripts through extensive alternative splicing. These include 29 unspliced forms and 28 spliced isoforms. Additional diversity arises from 5 alternative promoters, 9 non-overlapping final exons, and 9 verified polyadenylation sites. The resulting mRNAs vary in: 3’ end truncations, 5’ end truncations, presence/absence of 173 cassette exons, overlapping exons with different borders, and splicing versus retention of 3 introns^67^.

RBM20 regulates a subset of genes involved in developing the heart's muscles by modulating their mRNA alternative splicing. Titin, known to undergo extremely complex alternative splicing, is one of the RBM20’s targets. RBM20 specifically manipulates alternative splicing within the I-band of *TTN* pre-mRNA, which possesses the highest frequency of the alternative splicing process. It has been demonstrated that some alterations in the protein can produce pathogenic *TTN* isoforms, which are believed to lead to DCM^68^. Surprisingly, Khan et al.^69^, detected 80 distinct circRNAs among nearly a thousand from human hearts, indicating that the I-band of Titin is a hotspot region of circRNAs. Remarkably, the introns on each side of the back-spliced junctions were enriched in RBM20 binding sites, and the introns related to the *TTN* circRNAs had a five-fold higher frequency of RBM20 binding sites compared to a control set of introns. Studies on the RBP20 knock-out animals, and a cardiac sample of heterozygous RBM20 mutation carrier with substantially compromised synthesis of *TTN* circRNAs, both provided evidence that RBP20 is involved in the biogenesis of these *TTN* circRNAs^69^. Furthermore, the most recent study by Czubak et al.^70^, also found that Type 1 diabetes patients' human skeletal muscles included a significant amount of circRNAs primarily derived from the I-band of Titin. Titin has considerable interaction with other functional and structural proteins of sarcomeres. So, it is assumable that it has numerous binding sites for muscle-associated proteins and serves as an adhesion template for contractile machinery assembly in cardiac cells. So, it should be considered a dynamic and transformable molecule.

### The role of *TTN* variants in cardiomyopathies

Heterozygous mutations in *TTN* are commonly associated with cardiomyopathies and *TTN* has been reported as the most common gene involved in cardiomyopathies^71^. The mutations can be broadly classified into two categories, which are truncating or missense mutations. Truncating mutations lead to premature termination of Titin protein synthesis, resulting in either an altered protein or the loss of functional domains. In contrast, missense mutations result in the replacement of amino acids, potentially causing interference with the typical operation of the Titin protein^36^.

The ongoing inquiry into the exact molecular mechanisms by which *TTN* mutations lead to cardiomyopathies illuminates the intricate relationship between *TTN* mutations and various forms of cardiomyopathies. The haploinsufficiency model is a notable mechanism that proposes the presence of truncating mutations in one allele of the *TTN* gene results in a reduction in Titin expression, consequently inducing a functional deficit of Titin protein. The phenomenon mentioned above possesses the capability to disrupt the sarcomere assembly process, alter the mechanical properties of cardiac muscle cells, and prevent the heart's contractile function, leading to the manifestation of cardiomyopathy. Another proposed mechanism which even can be manifest in dominant pattern is missense mutations. This occurrence takes place when the mutated form of the Titin protein impairs the normal functioning of the unaltered Titin protein, leading to compromised assembly and operation of the sarcomere.

Moreover, it is plausible that *TTN* mutations may trigger aberrant splicing occurrences, leading to the production of deficient or abnormal Titin isoforms, thus playing a role in the pathogenesis of cardiomyopathy c. The bioinformatics analysis of reported variants in *TTN* related to cardiomyopathies has been shown in Table 1.

**Table 1:** Bioinformatics analysis of Pathogenic, Likely pathogenic, Unknown Significance reported variants in *TTN* related to cardiomyopathies.

No	Position on Chr. 2	HGVS DNA	HGVS Protein	Exon/Intron	dbSNP	ACMG	ClinVar	Mutation Taster	CADD	GERP	Reference
1	179391826	c.107889del	p.Lys35963AsnfsTer9	E.363	rs281864930	P	P	DC	76	5.79	^195^
2	179391848	c.107867T > C	p.Leu35956Pro	E.363	rs267607156	LP	LP	DC	35	6.17	^196^
3	179391875	c.107840T > A	p.Ile35947Asn	E.363	rs281864928	P	VUS	DC	34	4.9	^196^
4	179391915	c.107800G > T	p.Gly35934Ter	E.363	rs368277535	LP	VUS	DC	76	6.05	^197^
5	179391925	c.107780-107790delinsTGAAAGAAAAA	p.Glu35927-Trp35930delinsValLysGluLys	E.363	rs281864927	P	P	DC	65	4.87	^198^
6	179391925	c.107780-107781insTGAAAGAAAAA	p.Glu35927AspfsTer6	E.363	NA	LP	NA	PO	65	4.52	^196^
7	179391972	c.107743A > C	p.Thr35915Pro	E.363	NA	LP	NA	DC	32	6.06	^196^
8	179392207	c.107646del	p.Ser35883GlnfsTer10	E.362	NA	LP	NA	DC	75	5.76	^199^
9	179392218	c.107635C > T	p.Gln35879Ter	E.362	rs757082154	P	VUS	DC	75	4.87	^196^
10	179392275	c.107578C > T	p.Gln35860Ter	E.362	rs1009131948	P	LP/P	DC	73	3.75	^200^
11	179393000	c.107377 + 1G > A	–	I.361	rs112188483	P	P/LP	NA	NA	4.96	^201^
12	179393027	c.107351del	p.Ser35784Ter	E.361	rs778765016	P	NA	DC	81	4.97	^202^
13	179393094	c.107284C > T	p.Arg35762Ter	E.361	rs1477669354	P	LP	DC	70	4.36	^203^
14	179393272	c.107208del	p.Phe35736LeufsTer15	E.360	NA	P	NA	DC	75	5.17	^204^
15	179393329	c.107149C > T	p.Gln35717Ter	E.360	rs369157062	P	NA	DC	81	5.56	^202^
16	179393480	c.106998dup	p.Ala35667SerfsTer6	E.360	rs1031891465	LP	NA	NA	65	4.56	^202^
17	179393500	c.106978C > T	p.Gln35660Ter	E.360	rs1687693219	P	NA	DC	81	5.56	^205^
18	179393519	c.106959T > A	p.Tyr35653Ter	E.360	rs369450212	LP	NA	DC	41	− 7.15	^202,206^
19	179393524	c.106954C > T	p.Arg35652Ter	E.360	rs565675340	P	P	DC	70	− 3.94	^207^
20	179393564	c.106914G > C	p.Trp35638Cys	E.360	rs758497512	LP	VUS	DC	35	5.55	^205^
21	179393709	c.106768dup	p.His35590ProfsTer2	E.360	NA	P	LP	NA	65	5.10	^202^
22	179393738	c.106740del	p.Ala35581GlnfsTer36	E.360	NA	P	LP	DC	75	5.53	^202^
23	179393845	c.106668del	p.Lys35556AsnfsTer6	E.360	rs587776772	P	P	DC	75	2.76	^208^
24	178529118	c.106632-106633del	p.Leu35545LysfsTer3	E.360	NA	P	NA	DC	9.91	1.97	^204^
25	179393849	c.106629del	p.Ala35544ProfsTer2	E.360	rs869312069	P	LP	DC	75	2.82	^202^
26	179393907	c.106571del	p.Lys35524ArgfsTer22	E.360	rs199469666	P	NA	DC	73	3.39	^208^
27	179394686	c.106531 + 1G > A	–	I.359	rs760915007	P	P	NA	NA	5.61	^209^
28	179394796	c.106422del	p.Phe35475SerfsTer3	E.359	NA	LP	NA	DC	72	− 0.80	^206^
29	179394967	c.106374 + 1del	–	I.358	rs763404256|	LP	VUS	NA	NA	5.13	^202^
30	179395292	c.106050del	p.Glu35351AsnfsTer54	E.358	NA	LP	NA	DC	74	− 10.5	^206^
31	179395323	c.106019del	p.Gly35340ValfsTer65	E.358	rs727504482	P	NA	DC	74	5.23	^210^
32	179395428	c.105910-105914del	p.Thr35304CysfsTer3	E.358	NA	P	NA	DC	73	3.24	^206^
33	179395510	c.105832C > T	p.Gln35278Ter	E.358	NA	LP	NA	DC	11.95	2.7	^211^
34	179395528	c.105814del	p.Thr35272HisfsTer21	E.358	rs759645441	LP	NA	DC	66	0.59	^202^
35	179395600	c.105739-105742dup	p.Lys35248SerfsTer2	E.358	rs866421715	LP	NA	NA	62	0.88	^202^
36	179395807	c.105528-105535del	p.Gln35176HisfsTer9	E.358	rs199469665	P	LP	DC	66	3.57	^212^
37	179395811	c.105523-105531del	p.His35175-Val35177del	E.358	NA	VUS	NA	PO	53	3.49	^199^
38	179395856	c.105486del	p.Trp35162CysfsTer8	E.358	rs1553485330	P	P	DC	66	4.78	^213^
39	179395919	c.105423C > A	p.Tyr35141Ter	E.358	NA	LP	NA	DC	64	− 4.25	^214^
40	179396571	c.104771C > A	p.Ser34924Ter	E.358	rs1559003939	P	LP	DC	75	5.56	^215^
41	179396675	c.104666-104667del	p.Pro34889ArgfsTer3	E.358	NA	P	LP	DC	66	− 0.66	^216^
42	179396929	c.104413C > T	p.Arg34805Ter	E.358	rs750519430	P	LP/P	DC	71	4.59	^217^
43	179397250	c.104092C > T	p.Arg34698Ter	E.358	rs727504184	P	LP	DC	79	4.19	^202,218^
44	179397397	c.103945C > T	p.Arg34649Ter	E.358	rs995029896	P	LP	DC	74	3.46	^219^
45	179397492	c.103850-103851insAAC	p.Lys34618AspfsTer2	E.358	NA	VUS	NA	PO	62	0.00	^210^
46	179397546	c.103796G > A	p.Arg34599Lys	E.358	rs1362778188	LP	NA	DC	35	5.80	^205^
47	179397637	c.103705A > T	p.Lys34569Ter	E.358	rs1553490574	P	LP	DC	75	5.94	^202^
48	179397824	c.103518del	p.Ala34507LeufsTer8	E.358	rs1553491220	P	LP	DC	66	− 4.55	^209^
49	179397934	c.103408G > T	p.Glu34470Ter	E.358	rs769023413	LP	VUS	DC	68	5.78	^202^
50	179397982	c.103360del	p.Glu34454AsnfsTer3	E.358	rs760768093	P	P	DC	66	3.87	^220^
51	179398245	c.103096-103097insSVAelement	–	E.358	rs1575266261	NA	LP	NA	NA	NA	^202^
52	179398266	c.103073-103076dup	p.Ser34359ArgfsTer2	E.358	NA	P	LP	NA	62	0.89	^202^
53	179398340	c.103002-103003insA	p.Ala34335SerfsTer7	E.358	NA	P	NA	DC	62	3.24	^205^
54	179398393	c.102949C > T	p.Gln34317Ter	E.358	rs397517787	P	LP	DC	75	5.5	^80^
55	179398396	c.102946del	p.Tyr34316ThrfsTer3	E.358	NA	P	NA	DC	66	4.28	^205^
56	179398410	c.102932C > G	p.Ser34311Ter	E.358	NA	LP	NA	DC	72	5.6	^221^
57	179398712	c.102630del	p.Val34211Ter	E.358	rs869312101	p	VUS	DC	66	4.82	^202^
58	179398819	c.102523C > T	p.Arg34175Ter	E.358	rs752697861	P	P	DC	13.12	4.23	^221^
59	179398833	c.102509G > A	p.Trp34170Ter	E.358	NA	P	NA	DC	73	5.38	^205^
60	179399071	c.102271C > T	p.Arg34091Trp	E.358	rs140319117	P	VUS	DC	35	4.82	^205^
61	179399128	c.102214T > A	p.Trp34072Arg	E.358	NA	LP	NA	DC	34	5.88	^204^
62	179399285	c.102057del	p.Asn34020ThrfsTer9	E.358	NA	P	LP	DC	66	− 2.96	^204^
63	179400115	c.101227C > T	p.Arg33743Ter	E.358	rs794729305	P	LP	DC	76	4.63	^222^
64	179400229	c.101113del	p.Ser33705LeufsTer4	E.358	NA	P	NA	DC	65	4.4	^213^
65	179400244	c.101098-101099insT	p.Asp33700ValfsTer13	E.358	rs869312122	P	LP	DC	62	5.59	^202^
66	179400320	c.101021-101022del	p.Arg33674IlefsTer4	E.358	rs869312087	P	LP	DC	65	3.01	^202^
67	179400405	c.100936-100937del	p.Val33646HisfsTer26	E.358	NA	LP	NA	DC	65	4.08	^205^
68	179400516	c.100826G > A	p.Arg33609Gln	E.358	rs771243505	VUS	VUS	DC	35	5.3	^223^
69	179400517	c.100825C > T	p.Arg33609Ter	E.358	rs1057518195	P	LP/P	DC	72	5.3	^224^
70	179400577	c.100766-1G > T	–	I.357	rs185589320	LP	NA	NA	NA	5.3	^202^
71	179400887	c.100587G > A	p.Trp33529Ter	E.357	rs1064793560	P	LP	DC	70	5.76	^225^
72	179400913	c.100558-100561dup	p.Gly33521AspfsTer25	E.357	rs1553501572	P	LP	NA	62	4.18	^213^
73	179401029	c.100445C > A	p.Ser33482Ter	E.357	rs869312086	P	LP	DC	77	5.76	^202^
74	179401230	c.100244C > T	p.Pro33415Leu	E.357	rs72648282	LP	VUS	DC	35	5.76	^226^
75	179402067	c.99865 + 2T > C	–	I.355	rs1453570860	P	NA	NA	NA	5.53	^199^
76	179403522	c.99034A > T	p.Lys33012Ter	E.354	rs771511344	P	LP	DC	72	5.71	^199^
77	179403562	c.98994del	p.Lys32998AsnfsTer63	E.354	rs727504535	P	P	DC	65	3.68	^222^
78	179403888	c.98774del	p.Gly32925ValfsTer56	E.353	NA	P	LP	DC	65	6.15	^210^
79	179404189	c.98603del	p.Phe32868SerfsTer11	E.352	NA	P	NA	DC	65	3.44	^201^
80	179404241	c.98551C > T	p.Arg32851Ter	E.352	rs553821887	P	VUS	DC	69	3.78	^202^
81	179404286	c.98506C > T	p.Arg32836Ter	E.352	rs869312085	P	LP	DC	72	4.88	^202^
82	179404492	c.98299-98300del	p.Arg32767GlyfsTer2	E.352	rs397517776	P	P	DC	65	4.91	^202^
83	179404493	c.98299del	p.Arg32767GlyfsTer26	E.352	rs772061676	P	LP	DC	65	3.65	^202^
84	179404524	c.98265-98268dup	p.His32757AsnfsTer4	E.352	rs869312067	P	LP	NA	62	5.02	^202^
85	179404687	c.98105del	p.Pro32702LeufsTer15	E.352	NA	P	NA	DC	65	6.17	^213^
86	179405030	c.97863G > A	p.Trp32621Ter	E.351	NA	LP	NA	DC	68	5.96	^201^
87	179406990	c.97492 + 1G > A	–	I.349	rs727505319	P	NA	NA	NA	6.17	^227^
88	179407385	c.97192 + 4A > G	–	I.348	rs370069759	VUS	VUS	NA	NA	4.4	^202^
89	179407531	c.97050dup	p.Glu32351ArgfsTer6	E.348	rs794729365	P	P	NA	62	5.27	^228^
90	179407808	c.96892C > T	p.Gln32298Ter	E.347	rs201108270	LP	VUS	DC	68	5.91	^202^
91	179408200	c.96500-96501insAGAATTC	p.Gly32168GlufsTer27	E.347	NA	P	NA	DC	61	6.03	^205^
92	179408240	c.96460dup	p.Thr32154AsnfsTer39	E.347	rs869312084	P	LP	NA	61	4.75	^202^
93	179408364	c.96336-96337insC	p.Lys32113GlnfsTer3	E.347	NA	P	NA	DC	61	5.32	^80^
94	179408990	c.95966del	p.Asn31989ThrfsTer2	E.345	rs72648265	P	LP	DC	64	6.17	^199^
95	179409084	c.95872C > T	p.Arg31958Ter	E.345	NA	P	LP	DC	69	5.23	^229^
96	179410544	c.95416 + 3–95416 + 4insCCT	–	I.343	NA	LP	NA	NA	NA	3.31	^199^
97	179410545	c.95415–95416 + 2del	–	I.343	rs769407533	P	LP	NA	NA	5.82	^202^
98	179410592	c.95371G > C	p.Gly31791Arg	E.343	NA	P	VUS	DC	31	5.82	^230^
99	179410605	c.95358C > G	p.Asn31786Lys	E.343	rs869320743	P	P	DC	31	4.95	^231^
100	179410622	c.95341C > T	p.Arg31781Ter	E.343	NA	P	NA	DC	69	2.95	^205^
101	179410768	c.95195C > T	p.Pro31732Leu	E.343	rs753334568	P	LP/P	DC	35	5.82	^231^
102	179410778	c.95185T > C	p.Trp31729Arg	E.343	rs869320741	LP	P	DC	34	5.82	^231^
103	179410799	c.95164C > T	p.Gln31722Ter	E.343	NA	P	NA	DC	66	4.95	^199^
104	179410829	c.95134T > C	p.Cys31712Arg	E.343	rs869320740	LP	P	DC	33	5.82	^231^
105	179411050	c.95008C > T	p.Arg31670Ter	E.342	rs1322596650	P	P	DC	68	4.78	^232^
106	179411199	c.94859T > G	p.Leu31620Ter	E.342	rs561946873	LP	NA	DC	70	6.03	^207^
107	179411200	c.94852-94858del	p.Ala31618TyrfsTer37	E.342	rs869312066	P	LP	DC	64	4.51	^202^
108	179411203	c.94855C > T	p.Arg31619Ter	E.342	rs869312121	P	LP	DC	68	2.36	^202^
109	179411339	c.94816C > T	p.Arg31606Ter	E.341	rs1060500435	P	LP	DC	69	1.72	^233^
110	179411593	c.94562dup	p.Thr31522AsnfsTer12	E.341	rs869312083	P	LP	NA	61	2.50	^202^
111	179411905	c.94344-94347del	p.Lys31448AsnfsTer8	E.340	rs727503546	P	P	DC	64	5.67	^234^
112	179411967	c.94285T > A	p.Trp31429Arg	E.340	NA	LP	NA	DC	35	6.03	^196^
113	179412186	c.94167del	p.Phe31389LeufsTer7	E.339	rs747837187	LP	NA	DC	64	5.26	^202^
114	179412199	c.94154C > G	p.Ser31385Ter	E.339	rs548010682	LP	NA	DC	72	6.03	^207^
115	179412246	c.94103-94107del	p.Ile31368SerfsTer34	E.339	rs769488730	P	P	DC	64	5.33	^199^
116	179412456	c.93897del	p.Phe31299LeufsTer14	E.339	rs397517758	P	P	DC	64	3.15	^80^
117	179412902	c.93451G > T	p.Glu31151Ter	E.339	NA	P	NA	DC	67	5.65	^199^
118	179413151	c.93202G > T	p.Glu31068Ter	E.339	NA	P	NA	DC	68	5.65	^205^
119	179413187	c.93166C > T	p.Arg31056Ter	E.339	rs72648250	P	LP/P	DC	69	5.65	^202^
120	179413477	c.92876G > A	p.Trp30959Ter	E.339	rs72648249	P	NA	DC	67	5.22	^199^
121	179413670	c.92683C > T	p.Asp30885SerfsTer30895Ter	E.339	rs869312065	P	LP	DC	16.84	5.3	^202^
122	179413694	c.92652-92659del	p.Asp30885SerfsTer3	E.339	rs1559175090	P	LP	DC	63	2.1	^224^
123	178549148	c.92478dup	p.Val30827SerfsTer22	E.339	NA	P	LP	NA	7.36	3.45	^235^
124	179414036	c.92317C > T	p.Arg30773Ter	E.339	rs794729301	P	LP/P	DC	68	3.79	^225^
125	179414065	c.92284-92288dup	p.Ser30763ArgfsTer7	E.339	rs756367933	P	VUS	NA	64	4.17	^202^
126	179414119	c.92234C > A	p.Ser30745Ter	E.339	NA	P	NA	DC	67	5.74	^205^
127	179414186	c.92167C > T	p.Pro30723Ser	E.339	rs758537709	P	VUS	DC	32	5.73	^213^
128	179414303	c.92146C > T	p.Gln30716Ter	E.338	NA	P	NA	DC	70	5.73	^205^
129	179414366	c.92083T > C	p.Ser30695Pro	E.338	rs768267695	LP	NA	DC	31	5.74	^236^
130	179414574	c.91875del	p.Pro30626GlnfsTer2	E.338	rs757451467	P	P	DC	63	4.82	^205^
131	179414812	c.91753T > G	p.Phe30585Val	E.337	rs1060500507	P	VUS	DC	34	5.74	^237^
132	179414850	c.91715dup	p.Asn30572LysfsTer16	E.337	rs779129892	P	VUS	NA	61	4.08	^202^
133	179415706	c.91551-91552del	p.Asp30519Ter	E.336	NA	P	NA	DC	63	3.18	^205^
134	179416527	c.91097-91100dup	p.Asn30367LysfsTer3	E.335	NA	P	NA	NA	61	5.07	^79^
135	179416849	c.90778dup	p.Tyr30260LeufsTer12	E.335	rs397517750	P	LP	NA	61	4.10	^199^
136	179416870	c.90757G > A	p.Gly30253Arg	E.335	–	P	NA	DC	35	5.9	^205^
137	179417040	c.90587del	p.Lys30196ArgfsTer94	E.335	rs397517749	P	LP	DC	63	6.06	^238^
138	179417257	c.90370G > T	p.Glu30124Ter	E.335	rs1553539995	P	LP	DC	67	5.76	^239^
139	179417305	c.90322-90323insT	p.Glu30108ValfsTer6	E.335	rs869312082	P	LP	DC	61	5.76	^202^
140	178552691	c.90208-90209insSVAelement	–	E.335	NA	NA	LP	NA	NA	NA	^202^
141	179417539	c.90087-90088del	p.Glu30029AspfsTer7	E.335	rs869312064	P	LP	DC	63	3.32	^202^
142	179417542	c.90085del	p.Glu30029LysfsTer11	E.335	NA	P	NA	DC	63	5.76	^238^
143	179417543	c.90084del	p.Glu30029LysfsTer11	E.335	NA	LP	NA	DC	63	-9.19	^199^
144	179417724	c.89900-89903del	p.Asn29967MetfsTer27	E.335	rs869312081	P	LP	DC	63	4.36	^202^
145	179417877	c.89750dup	p.Val29918SerfsTer3	E.335	rs869312063	P	LP	NA	63	3.12	^202^
146	179418418	c.89314G > T	p.Glu29772Ter	E.334	NA	P	P	DC	64	4.71	^240^
147	179418468	c.89265G > A	p.Trp29755Ter	E.334	rs1179247052	P	LP	DC	66	5.6	^225^
148	179418639	c.89197 + 2T > G	–	I.333	rs1575536935	P	LP	DC	NA	5.61	^241^
149	179418639	c.89197–89197 + 2del	–	I.333	rs397517741	P	LP	NA	NA	4.10	^80^
150	179418640	c.89197 + 1G > C	–	I.333	rs1131691873	P	LP	DC	NA	5.61	^225^
151	179418877	c.88961G > A	p.Trp29654Ter	E.333	NA	P	NA	DC	66	5.61	^205^
152	179419329	c.88745C > T	p.Ser29582Phe	E.332	NA	LP	NA	DC	35	5.66	^237^
153	179419370	c.88703-88704del	p.His29568LeufsTer7	E.332	rs794729360	P	P	DC	63	5.29	^242^
154	179419765	c.88421G > A	p.Trp29474Ter	E.331	rs869025546	P	LP	DC	66	5.66	^243^
155	179422099	c.87887-87890del	p.His29296ProfsTer104	E.329	rs869312120	P	LP	DC	63	5.77	^202^
156	179422273	c.87716del	p.Gly29239AspfsTer32	E.329	rs869312028	P	VUS	DC	63	5.56	^202^
157	179422457	c.87624C > A	p.Tyr29208Ter	E.328	rs772121356	P	LP	DC	66	0.93	^202^
158	179422552	c.87529A > T	p.Lys29177Ter	E.328	NA	LP	NA	DC	33	4.44	^201^
159	179422565	c.87516del	p.Tyr29173ThrfsTer24	E.328	rs727503552	P	LP	DC	63	-1.28	^199^
160	179422726	c.87355del	p.Ala29119LeufsTer17	E.328	rs794729356	P	P	DC	63	5.63	^244^
161	179422902	c.87179C > A	p.Ser29060Ter	E.328	NA	P	NA	DC	67	5.69	^205^
162	179423093	c.87093del	p.Pro29032LeufsTer8	E.327	NA	P	NA	DC	63	4.57	^205^
163	179423146	c.87040C > T	p.Arg29014Ter	E.327	rs776065839	P	P	DC	67	4.77	^209^
164	179423220	c.86967G > A	p.Trp28989Ter	E.327	rs869312062	P	LP	DC	66	5.76	^202^
165	179423314	c.86872dup	p.Ser28958LysfsTer10	E.327	NA	P	NA	NA	61	0.07	^79^
166	179424036	c.86821 + 2T > A	–	I.326	rs397517735	P	P	DC	NA	5.61	^199^
167	179424057	c.86799-86802del	p.Gly28936Ter	E.326	rs727504856	P	P	DC	63	1.24	^228^
168	179424114	c.86742-86745del	p.Tyr28915ThrfsTer22	E.326	rs1415420768	P	LP	DC	63	0.88	^225^
169	179424219	c.86640C > A	p.Tyr28880Ter	E.326	NA	P	LP	DC	65	3.92	^202^
170	179424219	c.86640delC	p.His28881ThrfsX2	E.326	rs794729298	P	LP	DC	63	3.92	^202^
171	179424399	c.86459-86460del	p.Ser28820TrpfsTer50	E.326	rs869312080	P	LP	DC	63	2.79	^202^
172	179424496	c.86363G > A	p.Trp28788Ter	E.326	rs1064793814	P	P	DC	66	5.87	^199^
173	179424743	c.86116C > T	p.Arg28706Ter	E.326	rs794729384	P	P	DC	64	2.05	^232^
174	179424783	c.86076dup	p.Ser28693IlefsTer2	E.326	rs1285329277	P	P	NA	61	0.61	^245^
175	179424844	c.86015G > A	p.Trp28672Ter	E.326	NA	P	NA	DC	66	5.87	^223^
176	179424968	c.85891del	p.Ala28631LeufsTer3	E.326	rs1575610911	P	LP	DC	63	6.08	^246^
177	179425091	c.85768C > T	p.Arg28590Ter	E.326	rs748689777	P	P	DC	65	2.95	^227^
178	179425207	c.85640-85652del	p.Pro28547GlnfsTer12	E.326	rs762286447	P	LP	DC	63	4.12	^205^
179	179425598	c.85261-85262insAlu	–	E.326	NA	P	LP	NA	NA	NA	^247^
180	179425708	c.85151G > A	p.Arg28384Gln	E.326	rs1465916943	LP	NA	DC	34	5.09	^223^
181	179425709	c.85150C > T	p.Arg28384Ter	E.326	NA	P	LP	DC	65	3.09	^205^
182	179425748	c.85109-85111del	p.Lys28370-Ala28371delinsThr	E.326	NA	P	NA	DC	50	4.99	^223^
183	179425769	c.85090C > T	p.Arg28364Ter	E.326	rs770038577	P	LP/P	DC	66	5.09	^202^
184	179425848	c.85008-85011del	p.Glu28338HisfsTer9	E.326	rs869312100	P	VUS	DC	62	0.90	^202^
185	179426041	c.84819G > A	p.Trp28273Ter	E.326	rs72648222	P	P	DC	66	5.78	^199^
186	179426302	c.84557dup	p.Ile28187AsnfsTer6	E.326	rs1553564589	P	LP	NA	61	2.28	^80^
187	179426383	c.84476del	p.Gly28159ValfsTer15	E.326	rs1553564694	P	LP	DC	62	5.56	^80^
188	179426471	c.84388del	p.Cys28130ValfsTer44	E.326	NA	P	NA	DC	62	− 0.12	^205^
189	179426483	c.84376C > T	p.Gln28126Ter	E.326	rs869312119	P	LP	DC	66	5.22	^202^
190	179426940	c.83919del	p.Asn27973LysfsTer2	E.326	NA	LP	NA	DC	62	− 1.64	^223^
191	179427344	c.83515C > T	p.Arg27839Ter	E.326	rs869312118	P	P	DC	67	5.76	^202^
192	179427362	c.83497G > T	p.Gly27833Ter	E.326	NA	P	P	DC	66	4.87	^199^
193	179428087	c.82772G > A	p.Trp27591Ter	E.326	NA	P	NA	DC	66	5.85	^205^
194	179428202	c.82657G > T	p.Gly27553Ter	E.326	rs869178171	P	P	DC	65	4.96	^248^
195	179428256	c.82603A > G	p.Thr27535Ala	E.326	rs775733174	P	NA	DC	24	4.8	^236^
196	179428346	c.82513del	p.Ile27505PhefsTer20	E.326	rs869312060	P	LP	DC	62	0.86	^202^
197	179428522	c.82337C > T	p.Ala27446Val	E.326	rs780558473	LP	NA	DC	34	5.97	^249^
198	179428586	c.82273C > T	p.Gln27425Ter	E.326	rs371332011	P	LP	DC	65	5.97	^202^
199	179428871	c.81988C > T	p.Gln27330Ter	E.326	rs72648222	P	NA	DC	65	6.07	^199^
200	179428916	c.81943G > T	p.Glu27315Ter	E.326	rs373533040	P	LP	DC	66	6.07	^202^
201	179428920	c.81942del	p.Glu27315AsnfsTer35	E.326	NA	LP	NA	DC	62	0.49	^223^
202	179428980	c.81878-81879del	p.Phe27293CysfsTer3	E.326	rs727504660	P	P	DC	62	3.73	^199^
203	179429341	c.81518del	p.Pro27173HisfsTer17	E.326	rs869312079	P	LP	DC	62	4.63	^202^
204	179429515	c.81340-81344del	p.Lys27114GlnfsTer9	E.326	rs886038928	P	LP	DC	62	4.1	^250^
205	179429538	c.81321C > G	p.Tyr27107Ter	E.326	rs557312035	P	P	DC	64	4.22	^202^
206	179429590	c.81262-81269del	p.Gln27088CysfsTer5	E.326	rs869312059	P	LP	DC	62	4.82	^202^
207	179429862	c.80997-81012del	p.Tyr26999Ter	E.326	rs727503559	P	LP	DC	64	2.08	^251^
208	179430143	c.80716C > T	p.Arg26906Ter	E.326	rs727505284	P	P	DC	64	3.71	^252^
209	179430224	c.80635C > T	p.Gln26879Ter	E.326	rs79926414	LP	VUS	DC	65	5.49	^202^
210	179430320	c.80539C > T	p.Gln26847Ter	E.326	rs561152891	P	NA	DC	65	4.59	^243^
211	179430345	c.80514del	p.Val26839LeufsTer5	E.326	NA	P	P	DC	62	1.22	^199^
212	179430692	c.80167C > T	p.Arg26723Cys	E.326	rs1412497882	LP	VUS	DC	35	4.92	^223^
213	179430807	c.80052del	p.Gly26685AspfsTer11	E.326	NA	LP	NA	DC	62	3.32	^223^
214	179431048	c.79809-79811del	p.Val26604del	E.326	rs776591304	VUS	NA	PO	48	0.24	^223^
215	179431175	c.79684C > T	p.Arg26562Ter	E.326	rs869025545	P	LP	DC	65	4.03	^253^
216	179431293	c.79566T > A	p.Tyr26522Ter	E.326	NA	LP	NA	DC	62	− 2.28	^205^
217	179431416	c.79443del	p.Cys26482ValfsTer16	E.326	NA	P	NA	DC	62	2.22	^243^
218	179431868	c.78991C > T	p.Arg26331Ter	E.326	rs779996703	P	P	DC	65	1.45	^254^
219	179431880	c.78979C > T	p.Arg26327Ter	E.326	rs1419374180	P	LP	DC	65	0.75	^232^
220	179432352	c.78507del	p.Gly26170ValfsTer3	E.326	rs869312058	P	LP	DC	62	3.06	^202^
221	179432357	c.78502G > A	p.Ala26168Thr	E.326	NA	LP	NA	DC	28.7	5.75	^199^
222	179432675	c.78184G > T	p.Glu26062Ter	E.326	rs869312057	P	LP	DC	64	5.58	^202^
223	179432681	c.78178G > T	p.Glu26060Ter	E.326	rs794729289	P	P	DC	64	5.58	^225^
224	179432761	c.78095-78098del	p.Arg26032ThrfsTer41	E.326	rs869312117	P	LP	DC	62	4.37	^202^
225	179433095	c.77764C > T	p.Gln25922Ter	E.326	rs794729288	P	VUS	DC	65	5	^210^
226	179433197	c.77646-77662delinsAGA	p.Ile25883AspfsTer3	E.326	rs794729345	P	LP	DC	11.72	3.33	^199^
227	179433210	c.77647-77649del	p.Ile25883del	E.326	NA	LP	P	DC	48	1.91	^199^
228	179433274	c.77585del	p.Lys25862ArgfsTer25	E.326	NA	P	NA	DC	62	6.03	^205^
229	179433407	c.77452G > T	p.Glu25818Ter	E.326	NA	P	P	DC	63	6.03	^205^
230	179433438	c.77421dup	p.Ser25808GlnfsTer19	E.326	rs730880343	P	LP	NA	61	3.64	^80^
231	179433632	c.77227G > T	p.Glu25743Ter	E.326	rs765997807	P	LP	DC	64	5.74	^223^
232	179433630	c.77226-77229del	p.Ser25742ArgfsTer9	E.326	NA	P	NA	DC	61	3.86	^196^
233	179433665	c.77194C > T	p.Gln25732Ter	E.326	NA	P	NA	DC	64	5.74	^243^
234	179433714	c.77145dup	p.Ser25716LeufsTer8	E.326	rs1205409465	P	LP	NA	60	3.91	^225^
235	179433758	c.77101-77102insT	p.Pro25701LeufsTer9	E.326	NA	P	NA	DC	60	5.83	^199^
236	179433759	c.77100dup	p.Pro25701ThrfsTer9	E.326	rs794729343	P	P	NA	60	3.71	^255^
237	179433781	c.77077-77078delATinsGA	p.Ile25693Asp	E.326	NA	LP	NA	DC	60	2.62	^256^
238	179434010	c.76849-76850insGT	p.Ser25617CysfsTer18	E.326	NA	P	NA	DC	60	3.76	^243^
239	179434060	c.76790-76799del	p.Arg25597ThrfsTer9	E.326	NA	P	NA	DC	61	4.08	^79^
240	179434161	c.76697-76698del	p.Leu25566ArgfsTer3	E.326	NA	P	NA	DC	61	2.12	^199^
241	179434463	c.76393-76396del	p.Asn25465Ter	E.326	rs727504483	P	LP	DC	59	2.75	^210^
242	179434473	c.76383-76386del	p.Asn25462LysfsTer4	E.326	rs869312078	P	LP	DC	61	3.78	^202^
243	179434486	c.76373del	p.Pro25458GlnfsTer9	E.326	rs869025553	P	P	DC	60	5.02	^243^
244	179434743	c.76116-76117insA	p.His25373ThrfsTer4	E.326	rs869312077	P	LP	DC	61	3.03	^202^
245	179435035	c.75824A > G	p.Tyr25275Cys	E.326	NA	LP	NA	DC	34	5.87	^249^
246	179435223	c.75633-75636dup	p.Val25213CysfsTer25	E.326	rs1553603036	P	LP	NA	60	4.42	^224^
247	179435390	c.75469C > T	p.Arg25157Ter	E.326	rs1553603394	P	P	DC	64	0.01	^220^
248	179435609	c.75250C > T	p.Arg25084Ter	E.326	rs794729286	P	P	DC	64	4.74	^257^
249	179435628	c.75231T > A	p.Tyr25077Ter	E.326	NA	P	LP	DC	63	− 4.72	^227^
250	179435718	c.75138-75141del	p.Lys25046AsnfsTer8	E.326	rs794729340	P	P	DC	60	4.16	^258^
251	179435736	c.75123T > A	p.Tyr25041Ter	E.326	rs753526510	P	VUS	DC	62	− 0.24	^202^
252	179435976	c.74880-74883dup	p.Pro24962AsnfsTer9	E.326	rs869312116	P	LP	NA	60	3.48	^202^
253	179436177	c.74682C > A	p.Tyr24894Ter	E.326	NA	P	NA	DC	63	1.11	^223^
254	179436456	c.74403del	p.Asn24802MetfsTer20	E.326	NA	P	NA	DC	60	3.495	^227^
255	179436521	c.74338C > T	p.Arg24780Ter	E.326	rs794729285	P	P	DC	64	5.09	^202^
256	179436553	c.74306dup	p.Asn24769LysfsTer2	E.326	rs869312056	P	LP	NA	59	4.02	^202^
257	179437013	c.73846C > T	p.Arg24616Ter	E.326	rs794729284	P	P	DC	64	3.98	^259^
258	179437291	c.73568del	p.Pro24523HisfsTer4	E.326	rs1559415567	P	P	DC	59	3.58	^260^
259	179437750	c.73109G > A	p.Trp24370Ter	E.326	rs869312115	P	LP	DC	63	5.19	^202^
260	179438060	c.72799C > T	p.Gln24267Ter	E.326	NA	P	P	DC	63	4.17	^205^
261	179438190	c.72669del	p.Asp24224IlefsTer8	E.326	rs727504531	P	P	DC	59	− 3.04	^260^
262	179438873	c.71980-71986delGCATATGinsTA	p.Ala23994Ter	E.326	rs794729338	P	P	DC	58	4.05	^199^
263	179439257	c.71602C > T	p.Arg23868Ter	E.326	rs397517689	P	P	DC	64	2.44	^227^
264	179439359	c.71500C > T	p.Gln23834Ter	E.326	rs730880242	P	LP	DC	63	5.7	^243^
265	179439438	c.71421T > A	p.Tyr23807Ter	E.326	NA	LP	NA	DC	61	− 4.4	^205^
266	179439506	c.71353A > G	p.Thr23785Ala	E.326	rs765937279	P	NA	DC	26.9	5.6	^223^
267	179439852	c.71007dup	p.Gly23670ArgfsTer6	E.326	NA	P	NA	NA	59	3.79	^243^
268	179439881	c.70978C > T	p.Arg23660Ter	E.326	rs1553612386	P	P	DC	63	5.51	^243^
269	179439924	c.70935del	p.Ala23647LeufsTer19	E.326	NA	P	NA	DC	59	5.06	^205^
270	179439980	c.70879C > T	p.Gln23627Ter	E.326	rs1575799625	P	LP	DC	64	4.71	^203^
271	179440068	c.70791del	p.Gly23598GlufsTer8	E.326	rs869312076	P	LP	DC	58	5.02	^202^
272	179440084	c.70775del	p.Val23592GlyfsTer4	E.326	rs1216966174	LP	NA	DC	59	3.16	^202^
273	179440168	c.70690-70691dup	p.Thr23565SerfsTer5	E.326	NA	P	NA	NA	59	4.66	^199^
274	179440565	c.70294G > C	p.Val23432Leu	E.326	NA	VUS	NA	DC	32	5.76	^237^
275	179440697	c.70162C > T	p.Arg23388Ter	E.326	rs781540455	P	P	DC	63	2.78	^261^
276	179440982	c.69877G > T	p.Gly23293Ter	E.326	rs869312114	P	LP	DC	62	5.87	^202^
277	179440999	c.69860G > A	p.Trp23287Ter	E.326	NA	P	LP	DC	63	5.87	^248^
278	179441016	c.69843del	p.Val23282Ter	E.326	rs869312075	P	LP	DC	53	3.28	^202^
279	179441101	c.69758C > T	p.Thr23253Ile	E.326	NA	LP	NA	DC	31	5.74	^236^
280	179441300	c.69671del	p.Pro23224HisfsTer10	E.325	NA	P	NA	DC	54	4.37	^205^
281	179441341	c.69630C > A	p.Tyr23210Ter	E.325	rs777602537	P	LP	DC	62	− 5.08	^205^
282	179441449	c.69522T > G	p.Tyr23174Ter	E.325	NA	P	P	DC	63	0.22	^199^
283	179441479	c.69491-69492del	p.Val23164GlyfsTer2	E.325	rs869312113	P	LP	DC	42	− 5.32	^202^
284	179441510	c.69458-69461dup	p.Asn23154LysfsTer14	E.325	rs397517679	P	LP	NA	57	2.62	^80^
285	179441550	c.69421-69422insAAAAG	p.Gly23141GlufsTer38	E.325	rs1247353236	P	LP	PO	59	4.64	^225^
286	179441649	c.69412 + 1G > A	–	I.324	rs869312074	P	LP	DC	NA	5.72	^202^
287	179442329	c.68824 + 5G > C	–	I.323	rs749639627	VUS	VUS	DC	NA	5.79	^199^
288	179442329	c.68824G > A	p.Glu22942Lys	E.323	rs199506676	VUS	VUS	DC	24.8	4.08	^202^
289	179443336	c.68329 + 2–68329 + 3insTT	–	I.321	rs536078303	LP	VUS	NA	NA	5.39	^246^
290	179443339	c.68328A > G	p.Thr22776 =	E.321	rs1553619783	VUS	VUS	DC	43	5.78	^199^
291	179443889	c.67868T > C	p.Ile22623Thr	E.320	NA	LP	NA	DC	31	5.98	^262^
292	179444012	c.67745del	p.Val22582AlafsTer10	E.320	NA	P	NA	DC	57	5.68	^199^
293	179444052	c.67705-67706insLINE1	–	E.320-I.319	NA	P	LP	NA	NA	NA	^219^
294	179444405	c.67519C > T	p.Gln22507Ter	E.319	rs1559490694	P	LP	DC	62	5.78	^196^
295	179444429	c.67495C > T	p.Arg22499Ter	E.319	rs574660186	P	P	DC	63	4.63	^202^
296	179444577	c.67349-2A > C	–	I.318	rs753948675	P	P	DC	NA	5.10	^263^
297	179444661	c.67348 + 5G > A	–	I.318	rs765587170	VUS	VUS	PO	NA	3.7	^199^
298	179444666	c.67348C > T	p.Gln22450Ter	E.318	NA	P	P	DC	62	2.24	^264^
299	179444735	c.67279C > T	p.Arg22427Ter	E.318	rs1200988060	P	LP	DC	63	0.99	^265^
300	179444855	c.67159del	p.Ile22387Ter	E.318	rs869312092	LP	VUS	DC	54	4.48	^202^
301	179444925	c.67089del	p.Lys22364ArgfsTer24	E.318	NA	P	NA	DC	56	1.07	^213^
302	179445166	c.66940G > T	p.Asp22314Tyr	E.317	rs768380109	LP	VUS	DC	24.6	5.25	^236^
303	179446219	c.66769 + 3–66769 + 7delAAGTAinsT	–	I.316	NA	LP	NA	NA	NA	4.29	^266^
304	179446300	c.66695T > A	p.Val22232Glu	E.316	NA	LP	NA	DC	31	5.41	^204^
305	179446471	c.66523-66524del	p.Leu22175IlefsTer8	E.316	rs866120156	P	NA	DC	52	2.96	^202^
306	179447667	c.65860-65863dup	p.Asp21955ValfsTer3	E313 –I.313	NA	P	NA	NA	57	3.88	^229^
307	179447693	c.65837C > G	p.Ser21946Ter	E.313	rs775504996	P	NA	DC	63	5.02	^267^
308	179448411	c.65498G > C	p.Arg21833Thr	E.312	NA	VUS	NA	DC	24.7	5.14	^205^
309	179448433	c.65476G > T	p.Glu21826Ter	E.312	rs763824247	P	LP	DC	63	6.02	^202^
310	179449208	c.65070del	p.Ile21691LeufsTer5	E.311	NA	P	NA	DC	57	4.15	^199^
311	179449453	c.64915C > T	p.Arg21639Ter	E.310	rs1432889079	P	LP	DC	63	4.3	^242^
312	179450018	c.64453C > T	p.Arg21485Ter	E.309	rs768345594	P	LP	DC	62	5.25	^202^
313	179451443	c.64185del	p.Ala21396LeufsTer26	E.308	NA	LP	NA	DC	56	− 10	^205^
314	179452145	c.63794-1G > A	–	I.306	rs2049262622	P	LP	DC	NA	5.98	^268^
315	179452435	c.63601C > T	p.Arg21201Ter	E.306	rs764243269	P	P	DC	63	4.92	^202^
316	179453427	c.63025C > T	p.Arg21009Ter	E.304	rs368452607	P	LP	DC	62	5.27	^202^
317	179453720	c.62733G > A	p.Trp20911Ter	E.304	NA	P	NA	DC	63	6.07	^243^
318	179453730	c.62722C > T	p.Arg20908Ter	E.304	rs543860009	P	P	DC	62	− 3.88	^224^
319	179453946	c.62506C > T	p.Arg20836Ter	E.304	rs757231565	P	VUS	DC	63	4.14	^202^
320	179454235	c.62217T > A	p.Tyr20739Ter	E.304	rs727503586	P	P	DC	62	2.63	^199^
321	179454531	c.61921C > T	p.Arg20641Ter	E.304	rs878854324	P	P	DC	63	5.2	^268^
322	179454576	c.61876C > T	p.Arg20626Ter	E.304	rs72646846	P	P	DC	62	5.17	^242^
323	179454770	c.61682C > G	p.Ser20561Ter	E.304	rs1114167324	P	LP	DC	62	4.21	^244^
324	179454784	c.61668del	p.His20557MetfsTer20	E.304	NA	LP	NA	DC	54	− 0.84	^223^
325	179454957	c.61495C > T	p.Arg20499Ter	E.304	rs869312112	P	LP	DC	62	3.97	^224^
326	179455112	c.61339del	p.Ile20447Ter	E.304	rs1576086839	P	LP	DC	52	6.11	^243^
327	179455162	c.61290T > A	p.Cys20430Ter	E.304	NA	P	NA	DC	63	6.11	^199^
328	179455521	c.60931C > T	p.Arg20311Ter	E.304	rs869312055	P	LP	DC	62	5.23	^202^
329	179455598	c.60854-60855insG	p.Asn20286LysfsTer13	E.304	NA	P	LP	DC	55	5.535	^205^
330	179455719	c.60733C > T	p.Arg20245Ter	E.304	rs1057522256	P	P	DC	62	4.26	^205^
331	179455726	c.60726T > A	p.Tyr20242Ter	E.304	rs145423907	LP	NA	DC	61	− 1.83	^202^
332	179455780	c.60672del	p.Gly20225GlufsTer7	E.304	NA	P	NA	DC	55	0.045	^205^
333	179456553	c.59993G > A	p.Trp19998Ter	E.303	NA	P	NA	DC	62	6.16	^79^
334	179456704	c.59926 + 1G > A	–	I.302	rs553526525	P	P	DC	NA	6.16	^269^
335	179456766	c.59865-59866insA	p.Gln19956ThrfsTer9	E.302	NA	P	NA	DC	45	4.98	^205^
336	179456783	c.59848C > T	p.Arg19950Ter	E.302	rs1559598775	P	LP	DC	63	5.16	^253^
337	179457005	c.59627-1G > A	–	I.301	rs869312073	P	LP	DC	NA	6.03	^202^
338	179457273	c.59460G > A	p.Trp19820Ter	E.301	rs1250461669	P	LP	DC	62	6.03	^225^
339	179457321	c.59411dup	p.Arg19805LysfsTer3	E.301	rs755261062	P	LP	NA	54	2.46	^202^
340	179457380	c.59352del	p.Glu19785SerfsTer2	E.301	rs869312111	P	LP	DC	53	5.01	^202^
341	179457644	c.59201-59202del	p.Pro19734ArgfsTer5	E.300	rs752948913	P	LP	DC	52	4.85	^257^
342	179457977	c.58958G > C	p.Arg19653Pro	E.299	NA	LP	NA	DC	32	6.16	^205^
343	179458080	c.58855del	p.Glu19619LysfsTer27	E.299	NA	LP	NA	DC	52	6.16	^199^
344	179458083	c.58852dup	p.Arg19618LysfsTer6	E.299	NA	LP	NA	NA	54	1.43	^205^
345	179458293	c.58732 + 2T > C	–	I.298	rs869312054	P	LP	DC	NA	6.02	^202^
346	179458407	c.58620del	p.Val19541PhefsTer22	E.298	rs1576147786	P	LP	DC	52	5.63	^210^
347	179458459	c.58567-58568dup	p.Lys19524ValfsTer8	E.298	rs1553650442	P	P	NA	53	3.26	^234^
348	179458477	c.58550T > C	p.Ile19517Thr	E.298	rs72646838	VUS	VUS	DC	24.8	5.86	^226^
349	179458850	c.58270G > T	p.Glu19424Ter	E.297	rs72646837	P	P	DC	63	6.17	^199^
350	179458948	c.58172del	p.Asp19391AlafsTer45	E.297	rs869312072	P	LP	DC	52	5.03	^202^
351	179459155	c.58066dup	p.Glu19356GlyfsTer27	E.296	NA	LP	NA	NA	54	4.11	^199^
352	179459226	c.57995del	p.His19332ProfsTer18	E.296	rs397517633	P	LP	DC	52	6.17	^80^
353	179460233	c.57847 + 1G > A	–	I.295	rs397517631	LP	VUS	DC	NA	6.07	^80^
354	179460312	c.57769C > T	p.Arg19257Ter	E.295	rs794729275	P	LP	DC	62	5.08	^270^
355	179460320	c.57761A > G	p.Tyr19254Cys	E.295	NA	VUS	NA	DC	33	5.98	^10^
356	179460363	c.57718C > T	p.Arg19240Ter	E.295	rs2051361827	P	LP	DC	62	3.94	^79^
357	179460478	c.57603C > A	p.Cys19201Ter	E.295	rs1418030810	P	LP	DC	62	5.17	^225^
358	179462264	c.57544 + 1G > A	–	I.294	rs2052045274	P	LP	DC	NA	6.06	^202^
359	179462478	c.57331C > T	p.Arg19111Ter	E.294	rs72646831	P	P	DC	62	4.23	^228^
360	179462682	c.57215del	p.Gly19072GlufsTer12	E.293	rs397517628	P	LP	DC	54	5.87	^80^
361	179463603	c.56834del	p.Gly18945ValfsTer6	E.291	rs869312110	P	LP	DC	53	4.97	^202^
362	179463948	c.56572C > T	p.Arg18858Ter	E.290	rs745376275	P	LP	DC	62	3.19	^271^
363	179464342	c.56286T > A	p.Tyr18762Ter	E.289	NA	P	NA	DC	62	− 1.01	^205^
364	179464422	c.56206del	p.Thr18736ProfsTer8	E.289	rs869312109	P	LP	DC	49	4.5	^202^
365	179466193	c.55525-55531del	p.Asp18509SerfsTer29	E.287	rs869312052	P	LP	DC	50	4.37	^202^
366	179466263	c.55460-55461del	p.Lys18487SerfsTer3	E.287	rs1064796230	P	P	DC	49	4.96	^272^
367	179466466	c.55351C > T	p.Arg18451Ter	E.286	rs1440093502	P	P	DC	62	5.83	^205^
368	179466515	c.55303-1G > A	–	I.285	rs748369265	P	VUS	DC	NA	6.07	^202^
369	179466726	c.55269 + 3A > G	–	I. 284	rs72646820	P	NA	NA	NA	4.92	^199^
370	179468833	c.54581G > T	p.Gly18194Val	E.282	NA	LP	NA	DC	26.8	6.16	^205^
371	179469477	c.54339del	p.Glu18113AspfsTer10	E.281	rs796122911	P	LP	DC	51	4.33	^205^
372	179469738	c.54166C > T	p.Arg18056Ter	E.280	rs768431507	P	LP	DC	62	5.05	^272^
373	179469837	c.54067C > T	p.Arg18023Ter	E.280	rs1553682168	P	P	DC	62	4.83	^273^
374	179469882	c.54022G > A	p.Glu18008Lys	E.280	NA	P	NA	DC	23.9	5.74	^237^
375	179469986	c.53918del	p.Gly17973GlufsTer18	E.280	rs1486129583	P	P	DC	51	5.74	^199^
376	179470140	c.53881 + 1G > T	–	I.279	rs869312051	P	LP	DC	NA	5.63	^202^
377	179470359	c.53656-53663del	p.Pro17886Ter	E.279	NA	P	NA	DC	49	3.10	^205^
378	179471841	c.53488G > T	p.Gly17830Ter	E.278	rs759231562	P	LP	DC	62	5.35	^202^
379	179471975	c.53355G > A	p.Trp17785Ter	E.278	rs794729273	P	P	DC	62	5.99	^274^
380	179472042	c.53288-1G > C	–	I.277	rs1553685927	P	LP	DC	NA	5.99	^199^
381	179472127	c.53287 + 1G > T	–	I.277	rs1064794266	P	VUS	DC	NA	5.99	^199^
382	179472156	c.53259del	p.Lys17753AsnfsTer7	E.277	rs1389777522	P	LP	DC	48	5.19	^205^
383	179472209	c.53206C > T	p.Arg17736Ter	E.277	rs571702144	P	LP	DC	62	4.84	^275^
384	179472611	c.52903C > T	p.Arg17635Ter	E.276	NA	P	LP	DC	62	5.16	^276^
385	179473206	c.52406-2A > C	–	I.274	rs753798236	P	LP	DC	NA	5.72	^199^
386	179473427	c.52311-52312insTTGA	p.Gly17438LeufsTer12	E.274	NA	P	NA	DC	46	4.90	^205^
387	179473511	c.52223-52227dup	p.Asp17410ArgfsTer25	E.274	rs869312050	P	LP	NA	48	5.29	^202^
388	179473610	c.52128del	p.Phe17376LeufsTer27	E.274	rs869312095	LP	VUS	DC	49	3.55	^202^
389	179474002	c.52035-52036insTT	p.Leu17346PhefsTer4	E.273	rs869312049	P	LP	DC	51	2.55	^202^
390	179474121	c.51913-51916del	p.Lys17305ValfsTer13	E.273	rs747513278	P	LP	DC	50	1.81	^79^
391	179474220	c.51817G > T	p.Gly17273Ter	E.273	NA	P	NA	DC	61	5.85	^205^
392	179474816	c.51436 + 1G > A	–	I.271	rs761807131	P	P	DC	NA	5.48	^244^
393	179474817	c.51436C > T	p.Gln17146Ter	E.271	rs906494713	P	P	DC	62	5.48	^224^
394	179474936	c.51317G > A	p.Trp17106Ter	E.271	NA	P	NA	DC	61	5.48	^199^
395	179476484	c.50551 + 1G > A	–	I.268	rs188050862	LP	NA	DC	NA	5.18	^202^
396	179476569	c.50467C > T	p.Gln16823Ter	E.268	NA	P	NA	DC	62	5.08	^205^
397	179477005	c.50247del	p.Phe16749LeufsTer15	E.266	rs869312071	P	LP	DC	56	2.6	^202^
398	179477082	c.50170C > T	p.Arg16724Ter	E.266	rs794729265	P	P	DC	62	2.83	^202^
399	179477226	c.50026G > T	p.Glu16676Ter	E.266	NA	P	NA	DC	62	5.71	^196^
400	179477886	c.49648 + 2del	–	I.264	rs727504851	P	P	NA	NA	5.95	^199^
401	179478553	c.49458G > A	p.Trp16486Ter	E.263	rs869312108	P	LP	DC	61	6.07	^202^
402	179478665	c.49346-1G > A	–	I.262	rs869312070	P	P	DC	NA	6.07	^202^
403	179478861	c.49263C > A	p.Tyr16421Ter	E.262	NA	P	P	DC	58	0.84	^205^
404	179478865	c.49259del	p.Glu16420GlyfsTer23	E.262	NA	LP	NA	DC	55	6.07	^199^
405	179478953	c.49171C > T	p.Arg16391Ter	E.262	rs570046043	P	LP	DC	59	3.38	^277^
406	179479481	c.48761-1G > C	–	I.260	rs876657665	P	LP	DC	NA	5.63	^80^
407	179480145	c.48527G > A	p.Trp16176Ter	E.259	rs869312048	P	LP	DC	61	5.96	^202^
408	179480423	c.48405T > A	p.Cys16135Ter	E.258	rs371722903	LP	NA	DC	61	4.62	^202^
409	179480446	c.48382-48383insT	p.Lys16128IlefsTer6	E.258	rs771146720	LP	NA	DC	49	5.76	^202^
410	179481235	c.48283C > T	p.Arg16095Ter	E.257	rs374140736	P	P	DC	61	3.9	^202^
411	179481846	c.47875 + 1G > A	–	I.255	rs869312047	P	LP	DC	NA	5.76	^202^
412	179482115	c.47697C > A	p.Cys15899Ter	E.254	rs373040154	P	LP	DC	59	2.18	^202^
413	179482120	c.47692C > T	p.Arg15898Ter	E.254	rs775186117	P	LP	DC	61	0.77	^202^
414	179482230	c.47582G > A	p.Ser15861Asn	E.254	NA	VUS	NA	DC	28.2	6.08	^236^
415	179482584	c.47494C > T	p.Arg15832Ter	E.253	rs751746401	P	P	DC	62	4.74	^232^
416	179482662	c.47416del	p.Asp15806IlefsTer4	E.253	NA	P	NA	DC	53	5.63	^205^
417	179483042	c.47142-47143dup	p.Glu15715ValfsTer19	E.252	rs869312107	P	LP	NA	56	4.12	^202^
418	179483495	c.46782C > A	p.Tyr15594Ter	E.251	rs397517587	P	LP	DC	60	4.5999	^202^
419	179483504	c.46773T > A	p.Tyr15591Ter	E.251	rs397517586	P	LP	DC	57	3.1199	^80^
420	179485012	c.46236C > A	p.Cys15412Ter	E.248	rs368200299	P	LP	DC	60	2.73	^202^
421	179485178	c.46069-46070del	p.Met15357ValfsTer4	E.248	rs397517584	P	LP	DC	51	5.0099	^80^
422	179485525	c.45812T > G	p.Leu15271Ter	E.247	rs869312046	P	LP	DC	60	5.83	^202^
423	179485581	c.45756dup	p.Tyr15253IlefsTer15	E.247	rs869312045	P	LP	NA	49	5.44	^202^
424	179485589	c.45732-45748del	p.Glu15245PhefsTer17	E.247	NA	P	NA	DC	60	4.23	^205^
425	179485878	c.45567C > A	p.Tyr15189Ter	E.246	NA	LP	NA	DC	48	-9.56	^278^
426	179485878	c.45566dup	p.Tyr15189Ter	E.246	NA	P	NA	DC	48	0.73	^218^
427	179486054	c.45391delA	p.Ile15131TyrfsTer46	E.246	rs869312091	LP	VUS	DC	61	3.71	^202^
428	179486229	c.45322C > T	p.Arg15108Ter	E.245	rs1060500405	P	VUS	DC	61	6.17	^243^
429	179486244	c.45307C > T	p.Arg15103Ter	E.245	rs397517580	P	VUS	DC	61	3.01	^80^
430	179487411	c.44899C > T	p.Arg14967Ter	E.243	rs727505350	P	VUS	DC	60	2.65	^205^
431	179487495	c.44816-1G > A	–	I.242	rs749705939	P	VUS	DC	NA	5.54	^210^
432	179489209	c.44798G > A	p.Cys14933Tyr	E.242	NA	LP	NA	DC	28	5.72	^279^
433	179490056	c.44492G > C	p.Gly14831Ala	E.241	NA	LP	NA	DC	27.3	5.95	^279^
434	179494088	c.44364del	p.Tyr14789ThrfsTer15	E.240	rs397517576	P	VUS	DC	54	0.52	^205^
435	179494967	c.44281 + 1G > A	–	I.239	rs771562210	P	VUS	DC	NA	6.04	^202^
436	179494968	c.44281C > T	p.Pro14761Ser	E.239	rs192766485	VUS	VUS	DC	25.2	6.04	^202^
437	179494977	c.44272C > T	p.Arg14758Ter	E.239	rs140743001	P	VUS	DC	61	3.14	^202^
438	179495983	c.43792del	p.Val14598Ter	E.237	rs869312044	P	LP	DC	49	3.81	^202^
439	179497082	c.43539-43540insA	p.Ala14514SerfsTer10	E.236	NA	P	NA	DC	45	5.51	^199^
440	179497414	c.43319G > A	p.Trp14440Ter	E.235	rs372663057	LP	VUS	DC	60	6.16	^202^
441	179498055	c.42947-2A > G	–	I.232	rs1553741357	P	VUS	DC	NA	6.17	^199^
442	179498176	c.42909-42910del	p.Cys14303TrpfsTer12	E.232	rs1114167333	P	LP	DC	56	4.69	^244^
443	179498592	c.42636del	p.Ala14213LeufsTer6	E.231	rs869312106	P	LP	DC	57	0.47	^202^
444	179500295	c.41756A > G	p.Asp13919Gly	E.227	NA	VUS	NA	DC	24.4	6.05	^237^
445	179500825	c.41473C > T	p.Arg13825Ter	E.226	rs869312043	P	VUS	DC	57	0.36	^202^
446	179500851	c.41447del	p.Gly13816AlafsTer18	E.226	rs869312042	P	LP	DC	47	5.8	^202^
447	179505267	c.40723 + 1G > T	–	I.221	rs371770198	LP	VUS	DC	NA	0.36	^202^
448	179506963	c.40558 + 1G > A	–	I.219	rs368219776	LP	VUS	DC	NA	5.55	^199^
449	179506964	c.40558G > C	p.Val13520Leu	E.219	rs587780488	P	VUS	DC	24.9	5.55	^199^
450	179514543	c.39895 + 1G > T	–	I.211	179514543	LP	VUS	DC	NA	5.58	^202^
451	179516234	c.39492dup	p.Glu13165Ter	E.207	NA	P	NA	DC	55	5.22	^213^
452	179516991	c.39211G > T	p.Val13071Phe	E.203	rs1334646153	LP	VUS	DC	22.2	3.64	^199^
453	179516996	c.39204-39206dup	p.Thr13069dup	E.203	NA	VUS	NA	NA	60	0.72	^210^
454	179517379	c.39043 + 1G > T	–	I.201	rs373516134	LP	NA	DC	NA	5.64	^202^
455	179517464	c.38960–3-38960-1del	–	I.200	rs773282707	LP	NA	DC	NA	5.64	^202^
456	179523240	c.37579-37582del	p.Lys12527HisfsTer419	E.184	NA	P	NA	DC	43	2.07	^266^
457	179526509	c.37262del	p.Lys12421SerfsTer526	E.180	rs867008501	LP	NA	DC	60	2.65	^202^
458	179532021	c.35739dup	p.Pro11914SerfsTer7	E.162	rs968544783	LP	VUS	NA	46	0.67	^202^
459	179532190	c.35692A > T	p.Arg11898Ter	E.161	rs188568710	LP	NA	DC	52	4.84	^202^
460	179535816	c.35308 + 1G > T	–	I.156	rs1423135750	P	VUS	DC	NA	5.92	^199^
461	179537361	c.34855 + 1G > A	–	I.153	rs377319699	LP	VUS	DC	NA	5.25	^202^
462	179542346	c.34291 + 2T > C	–	I.146	rs186084940	LP	NA	DC	NA	6.17	^202^
463	179542507	c.34132del	p.Leu11378TyrfsTer90	E.146	rs869025551	LP	VUS	DC	53	-0.01	^243^
464	179544666	c.33535del	p.Glu11179SerfsTer3	E.140	rs757135518	LP	NA	DC	47	3.86	^202^
465	179544980	c.33418 + 1G > A	–	I.139	rs746588865	LP	VUS	DC	NA	4.98	^202^
466	179547631	c.32888-1del	–	I.134	rs869312041	P	VUS	DC	NA	5.18	^202^
467	179549632	c.32554 + 1G > C	–	I.130	rs376018437	LP	VUS	DC	NA	5.81	^202^
468	179549717	c.32471-1G > A	–	I.129	rs371725574	P	VUS	DC	NA	5.81	^202^
469	179554062	c.31966A > T	p.Lys10656Ter	E.124	rs368775510	LP	NA	DC	40	2.7	^202^
470	179554624	c.31763-1G > A	–	I.121	rs202234172	P	VUS	DC	NA	5.29	^227^
471	179558336	c.31594G > T	p.Val10532Phe	E.119	rs763955552	VUS	VUS	DC	24	5.85	^202^
472	179558736	c.31427-1G > A	–	I.117	NA	P	NA	DC	NA	6.16	^199^
473	179559325	c.31426 + 1G > C	–	I.117	rs6749719	LP	VUS	DC	NA	6.07	^202^
474	179559557	c.31347del	p.Val10450TyrfsTer25	E.116	NA	P	NA	DC	61	5.26	^205^
475	179560998	c.30803-2A > G	–	I.113	rs869312089	LP	VUS	DC	NA	5.5	^202^
476	179563643	c.30683-2del	–	I.111	rs1553868981	LP	VUS	DC	NA	5.57	^202^
477	179566913	c.30484-30493del	p.Thr10162CysfsTer3	E.108	rs727504452	P	LP	DC	61	3.22	^199^
478	179567322	c.30292G > T	p.Glu10098Ter	E.107	NA	P	NA	DC	58	5.72	^227^
479	179569962	c.29543G > A	p.Arg9848Gln	E.103	rs773444238	VUS	NA	DC	24.4	5.82	^280^
480	179571370	c.29231G > A	p.Arg9744His	E.102	rs760305440	VUS	VUS	DC	27.4	6.1	^280^
481	179571652	c.29071A > T	p.Lys9691Ter	E.101	rs376189903	LP	NA	DC	60	6.14	^202^
482	179571661	c.29062del	p.Ala9688GlnfsTer7	E.101	rs869312040	P	LP	DC	51	5.05	^202^
483	179571683	c.29042-2A > C	–	I.100	rs6716782	P	VUS	DC	NA	6.16	^202^
484	179572327	c.28967dup	p.Asp9656GlufsTer8	E.100	NA	LP	NA	NA	42	3.43	^202^
485	179575947	c.28016dup	p.Pro9340AlafsTer23	E.97	rs954237155	LP	NA	NA	54	2.52	^202^
486	179577042	c.27607G > A	p.Glu9203Lys	E.95	rs769097909	VUS	VUS	DC	25.7	5.88	^202^
488	179580418	c.25723G > A	p.Gly8575Arg	E.89	rs397517517	VUS	VUS	DC	24.2	5.33	^205^
489	179582078	c.25383del	p.Lys8461AsnfsTer5	E.88	rs1452206214	LP	NA	DC	61	-1.14	^202^
500	179582856	c.24863-24877del	p.Asp8288-Ile8293delinsVal	E.86	NA	LP	NA	DC	60	3.55	^210^
490	179583072	c.24749-24761del	p.Gly8250ValfsTer8	E.85	NA	LP	VUS	DC	60	2.80	^199^
491	179583429	c.24498dup	p.Val8167CysfsTer13	E.84	rs1282574211	P	NA	NA	41	3.99	^243^
492	179583702	c.24227-2A > G	–	I.83	rs373060681	P	NA	DC	NA	5.71	^202^
493	179584983	c.23386C > T	p.Arg7796Ter	E.81	rs748111134	LP	VUS	DC	51	5.91	^202^
495	179585718	c.23014-2328del	p.Ser7672-Ser7676del	E.79	NA		NA	DC	61	2.29	^210^
496	179586600	c.22788-22790delCATinsG	p.Asp7596GlufsTer16	E.78	NA	LP	NA	DC	60	5.08	^266^
497	179587599	c.22027C > T	p.Gln7343Ter	E.76	rs886043434	P	VUS	DC	50	5.8	^196^
498	179587773	c.21961G > A	p.Glu7321Lys	E.75	NA	P	NA	DC	23.2	5.95	^210^
499	179588844	c.21142C > T	p.Arg7048Ter	E73	rs770579313	LP	VUS	DC	41	1.61	^202^
501	179590572	c.20477C > A	p.Ser6826Ter	E.70	NA	P	NA	DC	42	4.85	^214^
502	179591958	c.20134del	p.Asp6712IlefsTer5	E.69	NA	P	NA	DC	51	6.17	^199^
503	179596801	c.16895T > C	p.Ile5632Thr	E.57	rs727504971	VUS	VUS	DC	23.1	6.17	^262^
504	179597846	c.16057C > T	p.Arg5353Ter	E.55	rs267599069	P	VUS	DC	47	5.29	^281^
505	179597615	c.16288C > T	p.Arg5430Ter	E.55	rs772235481	P	VUS	DC	44	5.29	^282^
506	179598224	c.15796C > T	p.Arg5266Ter	E.54	rs372277017	P	VUS	DC	45	1.13	^202^
507	179598245	c.15776-1G > T	–	I.53	rs869312094	LP	VUS	DC	NA	5.86	^202^
509	179598437	c.15679del	p.Ile5227SerfsTer29	E.53	NA	LP	NA	DC	59	0.07	^202^
510	179599054	c.15496 + 1G > A	–	I.52	rs397517481	P	VUS	DC	NA	5.86	^210^
511	179599091	c.15460G > A	p.Gly5154Ser	E.52	rs772907723	VUS	NA	DC	25.5	5.86	^205^
512	179602835	c.14344-14345delAGinsGA	p.Ser4782Asp	E.49	NA	VUS	NA	DC	61	5.8	^97^
513	179602866	c.14314T > C	p.Cys4772Arg	E.49	NA	VUS	NA	DC	26.6	5.8	^205^
514	179603088	c.14093-1G > A	–	I.48	rs869312099	P	VUS	DC	NA	5.37	^202^
515	179603867	c.14092 + 1G > T	–	I.48	NA	P	NA	DC	NA	5.62	^205^
516	179603904	c.14056del	p.Thr4686GlnfsTer9	E.48	rs869312104	P	LP	DC	49	0.97	^202^
517	179604264	c.13696C > T	p.Gln4566Ter	E.48	rs775072385	LP	VUS	DC	36	4.95	^199^
518	179604345	c.13615-13616insT	p.Asn4539IlefsTer5	E.48	NA	LP	NA	DC	61	− 5.64	^205^
519	179604363	c.13597del	p.Glu4533LysfsTer38	E.48	NA	LP	NA	DC	55	3.84	^205^
520	179604368	c.13592C > G	p.Ser4531Ter	E.48	NA	P	P	DC	36	4.91	^199^
521	179604528	c.13432-13433insA	p.Cys4478Ter	E.48	NA	LP	NA	DC	56	4.66	^230^
522	179604852	c.13108C > T	p.Gln4370Ter	E.48	rs267607158	P	P	DC	35	5.02	^97^
523	179604950	c.13010del	p.Lys4337SerfsTer14	E.48	NA	P	NA	DC	49	3.46	^199^
524	179598224	c.15796C > T	p.Arg5266Ter	E.48	rs372277017	P	VUS	DC	45	1.13	^202^
525	179605203	c.12757C > T	p.Gln4253Ter	E.48	rs869312039	P	LP	DC	38	4.91	^202^
526	179605317	c.12643C > T	p.Gln4215Ter	E.48	rs368329612	LP	VUS	DC	35	2.51	^202^
527	179605373	c.12587C > A	p.Ser4196Ter	E.48	rs370912401	P	VUS	DC	35	3.39	^202^
528	179605482	c.12478del	p.Thr4160ProfsTer8	E.48	NA	P	VUS	DC	40	1.23	^205^
529	179605512	c.12438-12448del	p.Ser4147ThrfsTer20	E.48	rs1553939749	P	LP	DC	43	− 0.52	^244^
530	179605752	c.12208G > T	p.Glu4070Ter	E.48	rs397517830	P	LP	DC	36	4.73	^283^
531	179606008	c.11952C > A	p.Tyr3984Ter	E.48	NA	P	NA	DC	38	0.85	^205^
532	179606286	c.11674T > C	p.Cys3892Arg	E.48	NA	VUS	NA	DC	21.9	6.08	^205^
533	179606303	c.11657del	p.Asp3886ValfsTer22	E.48	rs397517826	P	VUS	DC	60	6.08	^80^
534	179606362	c.11598C > A	p.Tyr3866Ter	E.48	NA	P	NA	DC	37	6.08	^205^
535	179606445	c.11497-11515del	p.Met3833CysfsTer3	E.48	NA	P	VUS	DC	60	1.90	^205^
536	179612657	c.11311 + 5184_11311 + 5194dup	–	I.47	rs869312088	VUS	VUS	NA	NA	0.83	^202^
537	179611822	c.11312-5174del	–	I.47	rs869312097	VUS	VUS	DC	NA	3.71	^202^
538	179611814	c.11312-5166C > T	–	I.47	rs376396183	VUS	NA	DC	NA	1.64	^202^
539	179610598	c.11312-3950del	–	I.47	rs774991940	VUS	VUS	DC	NA	3.58	^202^
540	179612712	c.11311 + 5139del	–	I.47	rs750893661	VUS	NA	DC	NA	1.18	^202^
541	179616552	c.11311 + 1299T > A	–	I.47	rs1561044021	P	NA	DC	NA	3.52	^205^
542	179616684	c.11311 + 1167del	–	I.47	rs869312096	VUS	VUS	DC	NA	2.36	^202^
543	179613467	c.11311 + 4384dup	–	I.47	rs771985828	VUS	VUS	DC	NA	1.80	^202^
544	179613188	c.11311 + 4663del	–	I.47	rs781363456	VUS	VUS	DC	NA	3.13	^202^
545	179613422	c.11311 + 4429G > T	–	I.47	rs372994805|	VUS	VUS	DC	NA	4.68	^202^
546	179613717	c.11311 + 4134dup	–	I.47	rs768458450	VUS	VUS	NA	NA	3.61	^202^
547	179610611	c.11312-3963G > T	–	I.47	rs148430495	VUS	VUS	DC	NA	5.94	^202^
548	179614105	c.11311 + 3746C > G	–	I.47	rs763408700	LP	VUS	DC	NA	5.25	^202^
549	179610383	c.11312-3735G > T	–	I.47	rs143376837	VUS	VUS	DC	NA	6.17	^202^
550	179614541	c.11311 + 3310G > T	–	I.47	rs372772094	VUS	NA	DC	NA	4.95	^202^
551	179615375	c.11311 + 2476G > T	–	I.47	rs373480236	VUS	NA	DC	NA	4.66	^202^
552	179616345	c.11311 + 1506del	–	I.47	rs777963995	VUS	NA	DC	NA	2.31	^202^
553	179620948	c.11254 + 1G > C	–	I.46	rs192945689	LP	NA	DC	NA	2.21	^202^
554	179620947	c.11254 + 2T > C	–	I.46	rs199565715	LP	VUS	DC	NA	0.77	^202^
555	179621013	c.11190C > G	p.Tyr3730Ter	E.46	rs373667402	LP	VUS	DC	38	1.99	^202^
556	179621020	c.11183dup	p.Leu3729ThrfsTer9	E.46	rs778172350	P	VUS	NA	61	3.38	^202^
557	179621090	c.11113del	p.Arg3705AspfsTer2	E.46	rs746386040	LP	VUS	DC	59	6.16	^202^
558	179621351	c.10852C > T	p.Gln3618Ter	E46	rs779064556	LP	VUS	DC	39	4.31	^202^
559	179621404	c.10799C > A	p.Ser3600Ter	E.46	rs374300381	LP	VUS	DC	40	6.17	^202^
560	179622355	c.10592C > G	p.Ser3531Ter	E.45	rs767420661	LP	VUS	DC	38	5.12	^202^
561	179622472	c.10475-10476insAGAC	p.Lys3493AspfsTer10	E.45	NA	LP	NA	DC	60	5.57	^210^
562	179623709	c.10303 + 2T > C	–	I.44	rs371596417	P	VUS	DC	NA	6.03	^202^
563	179629492	c.9749-9750del	p.Val3250AlafsTer40	E.42	rs1445295628	LP	NA	DC	60	1.06	^202^
564	179629515	c.9727C > T	p.Gln3243Ter	E.42	rs869312093	LP	VUS	DC	38	5.69	^202^
565	179631234	c.9577C > T	p.Arg3193Ter	E.41	rs746115846	P	VUS	DC	36	0.59	^211^
566	179632509	c.9448C > T	p.Arg3150Ter	E.40	rs146572907	P	VUS	DC	43	5.11	^202^
567	179632576	c.9381C > A	p.Tyr3127Ter	E.40	NA	P	LP/P	DC	36	2.19	^205^
568	179632841	c.9205del	p.Val3069TyrfsTer23	E.39	NA	P	NA	DC	61	2.956	^243^
569	179632884	c.9164-2A > T	–	I.38	rs777369921	LP	VUS	DC	NA	5.73	^202^
570	179633403	c.9160G > C	p.Glu3054Gln	E.38	–	VUS	NA	DC	23.2	5.81	^249^
571	179633431	c.9132del	p.Ala3045GlnfsTer14	E.38	rs36059692	LP	NA	DC	52	3.46	^202^
572	179634417	c.8891-8892insC	p.Thr2965AspfsTer17	E.37	NA	P	NA	DC	59	4.49	^205^
573	179634544	c.8764G > T	p.Glu2922Ter	E.37	NA	P	NA	DC	38	5.93	^205^
574	179634621	c.8687C > T	p.Thr2896Ile	E.37	rs72647884	VUS	VUS	DC	27.1	6.06	^226^
575	179635166	c.8353G > T	p.Gly2785Ter	E.35	NA	P	NA	DC	36	5.19	^243^
576	179635211	c.8307-8308del	p.Ala2770HisfsTer4	E.35	rs869312037	P	VUS	DC	61	4.71	^202^
577	179636183	c.7871dup	p.Pro2625AlafsTer9	E.34	rs1553997502	LP	NA	NA	43	3.86	^202^
578	179638333	c.7450C > T	p.Gln2484Ter	E.32	NA	P	VUS	DC	37	5.82	^199^
579	179639171	c.6820C > T	p.Gln2274Ter	E.30	rs145649088	P	VUS	DC	36	− 1.22	^202^
580	179640236	c.6355G > T	p.Glu2119Ter	E.28	rs869312098	LP	VUS	DC	36	5.33	^202^
581	179640343	c.6248del	p.Arg2083LysfsTer56	E.28	rs72647879	P	NA	DC	61	3.54	^236^
582	179640343	c.6248G > T	p.Arg2083Ile	E.28	rs781676050	VUS	NA	DC	21.9	3.54	^236^
583	179640344	c.6247del	p.Arg2083GlufsTer56	E.28	NA	P	NA	DC	57	3.68	^199^
584	179640468	c.6123G > A	p.Trp2041Ter	E.28	179640468	LP	NA	DC	36	5.19	^202^
585	179640970	c.5622G > A	p.Trp1874Ter	E.28	rs777078420	P	NA	DC	38	5.09	^197^
586	179641014	c.5577G > C	p.Arg1859Ser	E.28	NA	VUS	NA	B	23.6	1.41	^284^
587	179641524	c.5067G > A	p.Trp1689Ter	E.28	rs375648277	P	NA	DC	37	5.33	^202^
588	179641962	c.4724-4728del	p.Met1575SerfsTer6	E.27	rs756433029	P	VUS	DC	60	4.81	^202^
589	179641976	c.4714C > T	p.Arg1572Ter	E.27	rs1554008881	P	VUS	DC	37	3.89	^203^
590	179643691	c.4118C > A	p.Ala1373Glu	E.24	NA	VUS	NA	DC	25	5.91	^205^
591	179644006	c.3913G > A	p.Gly1305Arg	E.23	NA	VUS	NA	DC	25.9	5.72	^205^
592	179644174	c.3742-3745del	p.Ser1248ProfsTer14	E.23	NA	P	LP	DC	61	5.48	^205^
593	179647331	c.3101-2A > T	–	I.18	rs1060500467	P	VUS	DC	NA	5.54	^199^
594	179647533	c.3100G > A	p.Val1034Met	E18	rs142951505	VUS	VUS	DC	24	6.17	^202^
595	179647588	c.3045C > G	p.Cys1015Trp	E.18	NA	VUS	NA	DC	25	4.09	^223^
596	179647599	c.3034C > T	p.Arg1012Ter	E.18	rs397517547	P	VUS	DC	36	3.03	^80^
597	179647707	c.2926T > C	p.Trp976Arg	E.18	rs267607155	P	LP	DC	24.5	6.17	^285^
598	179647707	c.2926T > A	p.Trp976Arg	E.18	rs267607155	P	NA	DC	24.5	6.17	^10^
599	179648447	c.2841G > T	p.Ser947 =	E.17	rs774074192	LP	VUS	DC	45	1.07	^202^
600	179649078	c.2494G > T	p.Ala832Ser	E.16	rs376133574	P	VUS	DC	22.3	5.52	^202^
601	179650574	c.2370 + 1G > T	–	I.14	rs375796806	LP	NA	DC	NA	4.99	^202^
602	179650717	c.2228C > T	p.Ala743Val	E.14	rs267607157	VUS	P	PO	19.42	5.3	^97^
603	179650808	c.2137C > T	p.Arg713Ter	E.14	rs727505277	P	VUS	DC	39	5.99	^205^
604	179658212	c.1455dup	p.Ala486SerfsTer26	E.9	rs758662735	P	NA	NA	60	3.36	^243^
605	179659281	c.1246-3del	–	I.7	NA	VUS	NA	DC	NA	1.44	^199^
606	179659646	c.1245 + 3A > G	–	I.7	rs757221300	LP	VUS	DC	NA	5.87	^202^
607	179613717	c.11311 + 4134dup	–	I.47	rs768458450	VUS	VUS	NA	NA	3.61	^202^
608	179664231	c.897-898insT	p.Thr300TyrfsTer23	E.6	NA	P	NA	DC	58	− 2.83	^205^
609	179664293	c.835C > T	p.Arg279Trp	E.6	rs138060032	LP	VUS	DC	24.5	4.82	^191^
610	179665172	c.533C > A	p.Ala178Asp	E.4	NA	LP	NA	DC	23.4	5.16	^286^
611	179665380	c.325C > T	p.Arg109Ter	E.4	rs150954246	LP	VUS	DC	38	3.8	^202^

#### Dilated cardiomyopathy

Idiopathic factors are just as significant in the pathophysiology of DCM as acquired variables (such as infections, poisons, or autoimmune diseases). Individuals harboring *TTN* mutations exhibit a higher susceptibility to developing DCM compared to other forms of the disease^36,72–74^. Idiopathic DCM, including familial and sporadic instances, has a genetic etiology, according to a vast number of studies^75,76^.

A review study by Chauveau et al.^26^ reported that Among the *TTN* mutations linked to DCM, 29 are categorized as nonsense mutations, with three of them occurring in the I-band, while the remaining 26 are located in the A-band. Additionally, 17 frameshift mutations are reported, with three in the I-band and 14 in the A-band. Furthermore, 18 mutations are predicted to affect *TTN* splicing *TTN* mutations, particularly truncating variants (*TTN*tv) in the A-band region and in exons that are highly utilized across the range of titin isoforms, have been shown in a number of studies to be strongly associated with the occurrence of DCM and its severity, accounting for the majority of cases^77–80^.

Although fewer *TTN*tv have been identified in pediatrics, a study by Fatkin et al.^81^ on the young population showed that the prevalence between adolescents and adults is similar, indicating that they need to have multiple clinical and genetic risk factors other than a single *TTN*tv to present with CDM. *TTN*tv accounts for 25% of familial cases and 18% of sporadic cases of idiopathic dilated cardiomyopathy^82^. The aforementioned *TTN*tv have demonstrated a remarkably low prevalence within the broader populace.

According to Fatkin et al. the prevalence of *TTN*tv is 20% among individuals with DCM, whereas only 0.5% of the general population carries this type of mutation^83,84^. The aforementioned data aligns with the results of Fang et al.^85^ survey, which indicated an overall prevalence rate of 17%. The survey also revealed that 23% of cases were familial, while 16% were sporadic. For example, mutations in the A-band are implicated as predominant genetic causes of DCM^86–88^.

An important question is how minor TTNtv carrier populations can avoid presenting with DCM. A convincing explanation comes from a study by Roberts et al.^77^ showing that the two major adult cardiac titin isoforms, N2BA and N2B, are responsible. These abundant full-length isoforms predominantly contain distal A-band exons, where most DCM-causing TTNtvs are located. However, mutations in proximal exons not present in all TTN transcripts do not cause DCM.

#### Hypertrophic cardiomyopathy

HCM is the most common inherited cardiomyopathy, frequently arising from sarcomere gene defects. Characterized by arrhythmias and heart failure symptoms due to left ventricular outflow obstruction, diastolic dysfunction, ischemia, or mitral regurgitation, HCM displays autosomal dominant inheritance. Mutations, predominantly missense, in one or more sarcomere genes underlie most cases of HCM. To date, over 1400 mutations have been identified in genes encoding primarily sarcomeric proteins^89^.

Due to the involvement of a vast range of mutations with distinctive penetrance, a comprehensive understanding of the pathophysiological mechanisms underlying the development of HCM in the presence of sarcomere-related gene mutations is still unfulfilling^90^. In a study conducted by Ingles et al.^91^ on 33 genes reported to have an association with HCM, only 8 genes (*MYBPC3*, *MYH7*, *TNNT2*, *TNNI3*, *TPM1*, *ACTC1*, *MYL2*, and *MYL3*) were shown to have a definitive impact on occurring HCM. It is estimated that around 30% of HCM patients have unidentified genes responsible for the condition.

The gene *MYBPC3*, which codes for cardiac myosin-binding protein C, is the most important gene in this process accounting for up to half of the mutations identified^92–94^. In the second place, *MYH7*, which is responsible for encoding the beta-myosin heavy chain, is present in approximately 15–25% of patients diagnosed with HCM^92,95^.

In comparison to other plausible etiologies of HCM, the presence of the *TTN* gene mutations exhibits a relatively low ranking. Several studies reported four *TTN* variants resulting in gain-of-function effects in HCM patients. Satoh et al.^96^ found a Z-line mutation (c.2219G > T, p.Arg740Leu) which increases alpha-actinin binding affinity. Two studies, similarly, reported a mutation in cardiac-specific N2B exon 49 [c.12347C > A, p.Ser4116Tyr] resulting in increased TTN binding to DRAL/FHL2^97,98^. The TTN/T-CARP interaction is reinforced by the presence of two mutations located in exons 103 and 104-N2A, c.29231G > A, p.Arg9744 (initially reported as p.Arg8500His) and c.29543G > A, p.Arg9848Gln (initially reported as p.Arg8604Gln), as reported by Arimura et al.^99^. Lopes et al., in a different study, reported 219 *TTN* variants in a population of unrelated HCM patients. Of those 87% coexisted with mutations in HCM-related sarcomere gene defects and only 13% were found isolated^26,100^. However, in a study on 90 HCM patients and their close relatives, the mutation screening revealed no clue of the *TTN* gene being involved in their pathogenesis^101^. Similarly, Martijn Bos et al.^102^ detected no *TTN* mutation in a group of 389 HCM patients.

#### Restrictive cardiomyopathy

Restrictive cardiomyopathy is a diverse collection of disorders that primarily affect the myocardium, with a lesser impact on the endocardium and sub-endocardium. It is characterized by increased stiffness of the ventricular walls leading to restricted ventricular filling, which consequently results in significant diastolic dysfunction, elevated end-diastolic pressure, and reduced ejection fraction in the advanced stages^103,104^.

The epidemiology of this disease is not well understood in the literature due to classification and etiology reporting difficulties, but RCM is surely the least common form of cardiomyopathies, representing 2% to 5% of cases^2,105^. There are a variety of diseases that can cause it, including infiltrative disorders like amyloidosis and sarcoidosis, non-infiltrative disorders like diabetes and scleroderma, storage disease, endomyocardial disease, and cardiotoxicity brought on by chemotherapy or radiotherapy^2^.

Numerous genes that encode non-sarcomeric, sarcomeric, and sarcomere-associated proteins have been shown to play a role in RCM occurrence and inheritance. Examples include the *TTR* gene variants (V122I; I68L; L111M; T60A; S23N; P24S; W41L; V30M; V20I) and *APOA1* gene in Amyloidosis; *GLA* gene in Fabry disease; GBA gene in Gaucher disease; *HAMP*, *HFE*, *HFE2*, *HJV*, *PNPLA3*, *SLC40A1*, *TfR2* genes in Hereditary hemochromatosis; *NPC1*, *NPC2* and *SMPD1* genes in Niemann-Pick disease; *AG3, CRYAB, DES, DNAJB6, FHL1, FLNC, LDB3,* and *MYOT* genes in Myofibrillar myopathies; *ABCC6* gene in Pseudoxanthoma elasticum; *ACTC, MHC, TNNT2, TNNI3, TNNC1, DES, MYH, MYL3*, and *CRYAB* genes in Sarcomeric protein disorders; *WRN* gene in Werner's syndrome; and *BMP5*, *BMP7* and *TAZ* genes in Endocardial fibroelastosis^1,2,106,107^.

The role of *TTN* variants in RCM is relatively unknown and more investigations are needed to illustrate this fact. In 2013, for the first time, Peled et al. discovered a novel missense mutation (c.50057A > G, p.Tyr16686Cys) in the intersection of the A and I regions of Titin (IA junction). This mutation was found to play a role in early-onset familial RCM, which affected six members of a family. They asserted that Titin determines the sarcomere's resting tension, and their study offers genetic proof of its critical significance in diastolic function.^36,108,109^. In another study, Kizawa et al.^110^ found another novel *TTN* missense mutation (c.22769C > A, p.P7590Q) in a young boy with neurofibromatosis type 1, which is thought to be responsible for RCM co-occurrence. This de novo mutation is also located at the IA junction.

#### Arrhythmogenic right ventricular cardiomyopathy (ARVC)

Arrhythmogenic cardiomyopathy (ACM), is a rare and potentially life-threatening heart muscle disease with a prevalence of approximately 1:1000 to 1:5000^111–113^. Although asymptomatic in most instances upon diagnosis, it is characterized by palpitations, atypical chest pain, and syncope caused by cardiac arrhythmia, mostly in the right ventricle, which leads to the term “arrhythmogenic right ventricular cardiomyopathy (ARVC)”^114–116^. This condition is characterized by the progressive replacement of the myocardium with fibrofatty tissue, a process that begins at the epicardium, turns into a regional wall motion abnormality, and eventually spreads throughout the myocardium, resulting in the development of ventricular dilation and multiple aneurysms^117–119^.

The primary etiology of ACM is attributed to mutations in genes that encode desmosomal proteins, mainly with an autosomal dominant pattern of inheritance and over 30 percent of cases being familial. *JUP*, *DSP*, *PKP2*, *DSG2*, and *DSC2* genes are the most probable to be involved. *LMNA* and *TMEM43* are two additional genes that have been linked to the nuclear envelope, and there are genes that are shared with other cardiomyopathies (such as *DES*, *PLN*, *TGFB3*, *TTN*, and *SCN5A*)^112,120–123^.

Several studies have been conducted on the role of *TTN* variations in the pathogenesis of ARVC. In one study by Taylor et al.^121^, eight novel *TTN* variants (c.C29453T, p.Thr2896Ile; c.A97341G, p.Tyr8031Cys; c.C106734T, p.His8848Tyr; c.T215598C, p.Ile16949Thr; c.G221380A, p.Ala18579Thr; c.G226177T, p.Ala19309Ser; c.C272848T, p.Pro30847Leu; c.T281801C, p.Met33291Thr) were identified in seven unrelated families with well-established ARVC. They claimed the most prominent variant was Thr2896Ile, showing strong segregation evidence. In another investigation on the phenotype-genotype relationship of ARVC in 39 families, Brun et al.^123^ found 13% of their studied population, had rare *TTN* variants (c.29453C > T, p.Thr2896Ile; 281801T > C, Ala18579Thr; c.221380AG > T, p.Met33291Thr; c.226177G > T, p.Ala19309Ser; c.97341G > A, p.Tyr8031Cys; c.272848C > T, p.Pro30847Leu). In the investigation of the levels of Novex variant expression in human hearts with cardiomyopathies, Chen et al.^124^ came to the conclusion that this factor was altered in cardiomyopathies such as DCM and ARVC.

### Other muscle disorders

Beyond cardiomyopathies, TTN mutations are implicated in numerous non-cardiac muscle disorders. According to Chauveau et al.^26^, 39 TTN mutations have been identified so far in four pure skeletal muscle myopathies: limb girdle muscular dystrophy type 2J (LGMD2J), late-onset autosomal dominant tibial muscular dystrophy (TMD), hereditary myopathy with early respiratory failure (HMERF), and congenital centronuclear myopathy (CNM). Additional conditions associated with TTN variants include early adult onset recessive distal titinopathy, early-onset myopathy with fatal cardiomyopathy, multi-minicore disease with heart disease, childhood-juvenile Emery-Dreifuss-like phenotype without cardiomyopathy, and adult-onset recessive proximal muscular dystrophy^125^.

### Frequent TTN-related molecules in cardiomyopathies

**Figure 4:**
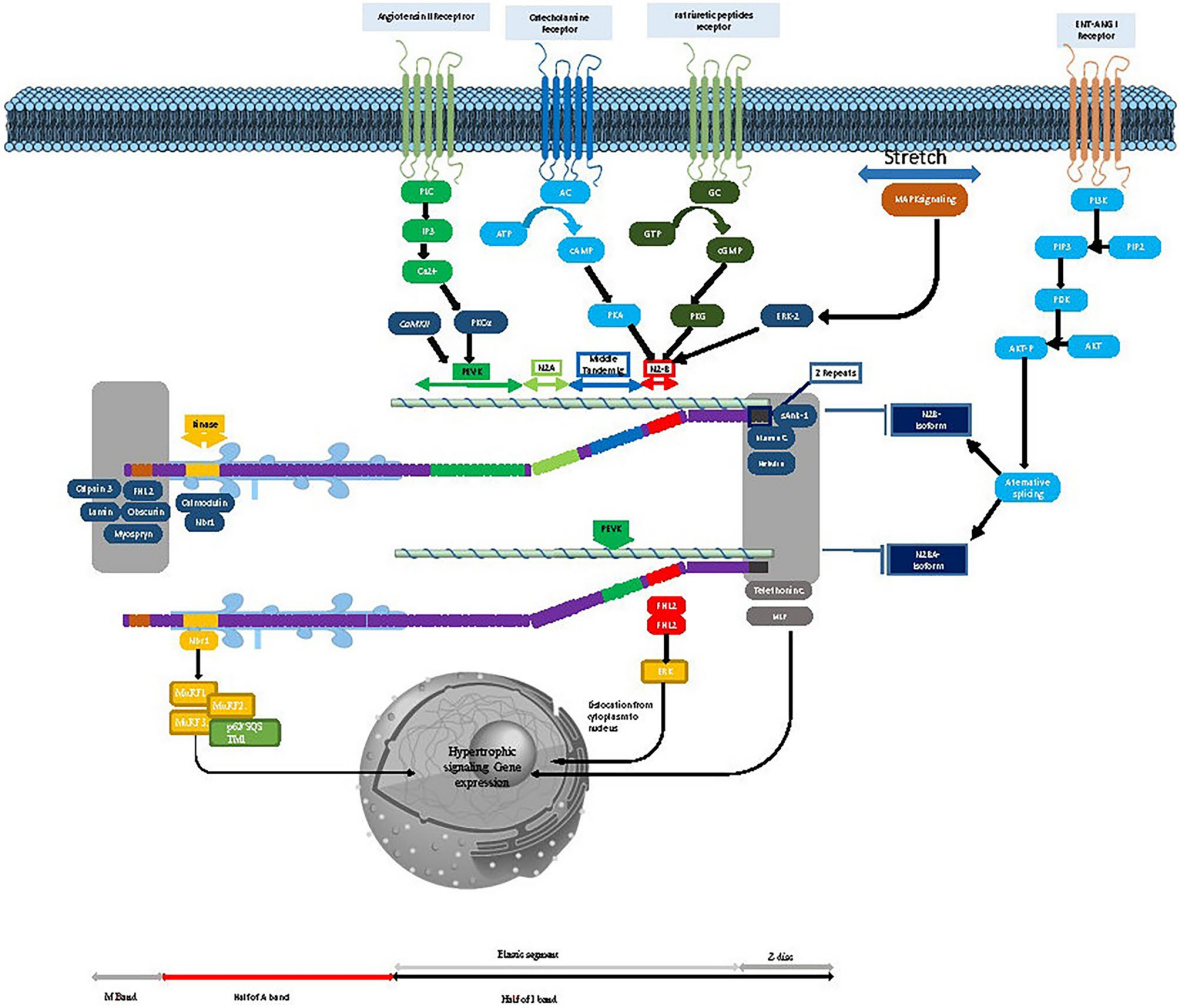
Illustration of the intricate signaling pathway implicated in the development of cardiomyopathy associated with Titin and other related proteins.

There are several molecules which play a considerable role in the signaling and function of Titin. In the present study, we evaluated their interaction with Titin and consider their interaction with Titin in the pathogenesis of cardiomyopathies (Fig. 4).

#### Calpain

Calpain, a family of Ca^2+^-dependent cytosolic cysteine proteases, plays a role in various cellular processes, including cell death and tissue remodeling^126^. It has been implicated in several cardiac conditions, including dilated cardiomyopathy, alcohol-related cardiomyopathy, chemotherapy-induced cardiomyopathy, arrhythmogenic cardiomyopathy, and diabetic cardiomyopathy^127–131^. Sustained over-expression of calpain-2, specifically in cardiomyocytes, induced age-dependent dilated cardiomyopathy in mice^127^.

#### MuRF1/2

Muscle ring finger (MuRF) proteins are muscle-specific ubiquitin E3 ligases that regulate the ubiquitin–proteasome system and modulate cardiac mass and function^132^. A study by Su et al.^133^ showed a higher prevalence of rare MuRF1 and MuRF2 variants in hypertrophic cardiomyopathy (HCM) patients compared to controls. HCM patients with these rare MuRF1/2 variants were younger and had greater maximum left ventricular wall thickness than those without the variants^133^.

#### ERK

ERK (Extracellular signal-regulated kinase) plays a central role in cardiac physiology and hypertrophy^134–136^. ERK signaling is implicated in various forms of cardiac hypertrophy and progression to heart failure^135^. Altered ERK activity has been linked to HCM^134^. ERKs are considered key regulators of cardiac hypertrophy since they are activated by most, if not all, stress stimuli known to induce hypertrophic growth^137^. Studies show that concurrently eliminating ERK1 and ERK2 in the heart leads to eccentric hypertrophy with chamber dilatation and cardiomyocyte elongation^136^.

#### NFAT

Nuclear factor of activated T-cells (NFAT) transcription factors are implicated in developing cardiac hypertrophy and heart failure^138^. Activation of NFAT signaling induces pathological remodeling of cardiomyocytes^139^. Inhibition of NFAT prevents maladaptive cardiac growth in response to stress stimuli^140^. Targeting NFAT signaling pathways may be therapeutic for specific cardiomyopathies^141,142^.

#### FHL1/2

Mutations in the four-and-a-half LIM domain proteins 1 and 2 (FHL1 and FHL2) are associated with reducing body myopathy and hypertrophic cardiomyopathy^143^. FHL1/2 are involved in sarcomere assembly and signaling and highly expressed in skeletal and cardiac muscle^144,145^. Abnormal FHL proteins cause structural defects in sarcomeres and impaired muscle contraction^146^. FHL1 mutations account for 8–10% of familial reducing body myopathy cases which can include cardiomyopathy^147,148^. Chu et al.^145^ reported FHL1 upregulation in Cardiac ventricles of two mouse models with cardiac hypertrophy and dilated cardiomyopathy.

#### MARP

Muscle ankyrin repeat proteins (MARPs), including CARP, Ankrd1/2, and DARP, are a family of ankyrin repeat proteins expressed in striated muscle that are induced by stress. MARPs play regulatory roles in the muscle stress response and hypertrophy pathogenesis^149^. Overexpression of CARP is linked to dilated cardiomyopathy in animal models^150^. In addition, Patients with hypertrophic, dilated, ischemic, and arrhythmogenic right ventricular cardiomyopathy are more likely to develop CARP upregulation^62,149,151,152^. Missense mutations in the Ankrd1 gene have recently been identified as the cause of dilated and hypertrophic cardiomyopathy in humans^99,149,153,154^. CARP modulation of gene expression may contribute to adverse ventricular remodeling in cardiomyopathies^155^.

#### Nbr1

Neighbor of BRCA1 gene 1 (Nbr1) is a cardiac-expressed protein involved in autophagy, protein degradation and sarcomere organization^156^. Several studies suggested role of Nbr1 overexpression in developing dilated cardiomyopathy^157–159^.

#### SRF

Serum response factor (SRF) is a transcription factor regulating cardiac gene expression important for adaptation to stress^160^. SRF inactivation in animal models causes dilated cardiomyopathy^160^. SRF likely controls genes involved in maintaining normal cardiac structure and function^161^. Alterations in SRF-dependent gene regulation may underlie some cardiomyopathies^162^.

#### MLP

Muscle LIM protein (MLP) is involved in mechanosensing and stretch response in cardiomyocytes^163^. MLP knockout mice develop dilated cardiomyopathy^164^. Loss of MLP leads to impaired myocyte stretch signaling and contraction^165^. MLP deficiency is implicated in some forms of familial dilated cardiomyopathy^166^.

#### MyBP-C

Myosin binding protein C (MyBP-C) is important for maintaining sarcomere structure and regulating muscle contraction^167^. Mutations in cardiac MyBP-C are the most common cause of hypertrophic cardiomyopathy^168^. Abnormal MyBP-C disrupts sarcomere function leading to reduced contractility and development of hypertrophy^169^.

#### Myomesin

Myomesin is a major component of the sarcomeric M-band involved in thick filament organization^170^. Myomesin mutations have been associated with hypertrophic and dilated cardiomyopathy in some patients^171^. Altered myomesin disrupts myofilament integrity and crosstalk resulting in cardiomyocyte damage^172^.

#### Sh2 domain

Src homology 2 (SH2) domains mediate protein–protein interactions in cell signaling cascades^173^. Mutations affecting SH2 domains of ZASP/Cypher proteins are linked to dilated cardiomyopathy^174^. Disruption of ZASP protein interactions likely impairs structural organization and signaling processes in cardiac muscle^175^.

#### Ras

Ras family small GTPases regulate growth and survival signaling^176^. Constitutively active mutant Ras expressed in mouse hearts causes dilated cardiomyopathy phenotype^177^. Hyperactive Ras leads to increased cell growth, altered metabolism and myocardial dysfunction^178^.

#### Raf

Raf kinases act downstream of Ras to activate MEK/ERK signaling involved in cell proliferation and differentiation^179^. Cardiac-specific expression of activated Raf in transgenic mice induces dilated cardiomyopathy^180^.

#### Alpha actinin

Alpha-actinin-2 (ACTN2) is the sole muscle isoform of α-actinin expressed in cardiac muscle^181^. Previous studies have shown that novel ACTN2 variants are associated with familial HCM^182^. Previous studies have shown that novel ACTN2 variants are associated with^181^. Mutations in ACTN2 have been linked to mild to moderate forms of HCM^181^. Disease modeling of an ACTN2 mutation has guided clinical therapy in HCM^183^. Genome-wide analyses have also demonstrated that ACTN2 mutations can cause HCM^184^.

#### Filamin C

In striated muscle, different forms of the Ank3 gene product (ankyrins-G) are produced due to tissue-specific alternative splicing. These ankyrins-G have a shared segment called the Obscurin/Titin-Binding-related Domain (OTBD), which is consistent across ankyrin genes and links obscurin and Titin to Ank1 gene products. Previously, it was suggested that the OTBD segment in ankyrins plays a unique role in muscle protein interactions. In recent studies, muscle proteins that can bind to the ankyrin-G OTBD were identified as plectin and filamin C, both crucial for muscle development and structure. These three proteins (ankyrin-G, plectin, and filamin C) are found together in skeletal muscle and are observed in the same regions (costameres) of adult muscle fibers^185^. Filamin C (FLNC) is an actin-binding cytoskeletal protein encoded by the FLNC gene, instrumental in maintaining sarcomeric integrity. While first identified as causative in myofibrillar myopathy, recent evidence reveals a key role for FLNC in cardiomyopathy pathogenesis. Truncated FLNC variants predominate in DCM and ARVC, while non-truncated forms are more common in hypertrophic cardiomyopathy and restrictive cardiomyopathy. The primary mechanisms underlying FLNC-associated cardiomyopathies are protein aggregation from non-truncating mutations and haploinsufficiency resulting from filamin C truncation^186^.

#### Nebulin

Members of the nebulin protein family, which includes nebulin, nebulette, LASP-1, LASP-2, and N-RAP, are diverse in size, expression pattern, and function, but they all bind to actin. While nebulin's presence in the heart is minimal, nebulette stands out for its heart-specific expression. Crucially, mutations in the nebulette gene have been linked to DCM. Transgenic mice with these mutations display symptoms that mirror this human heart condition^187^.

### Mechanosensory signaling mechanism of titin

Titin plays a crucial role in mechanosensing, which is the ability of cells to sense mechanical forces. When muscles undergo stretch or contraction, Titin is subjected to mechanical stress and strain. This mechanical deformation of Titin can trigger mechanotransduction pathways, converting mechanical signals into biochemical signals. These pathways involve the activation of various signaling molecules, including kinases, phosphatases, and transcription factors, leading to cellular responses such as gene expression changes, protein synthesis, and remodeling of the contractile apparatus^188^ (Fig. 4).

#### Z disk region

The Z-disc region of Titin consists of Z-repeats and Ig-domains Z1 and Z2, forming the very NH2-terminal end. Telethonin connects two Titin molecules from one sarcomere, which is essential for sarcomere integrity. Cardiac telethonin undergoes phosphorylation by various kinases and mutations in telethonin are linked to various cardiac cardiomyopthies. Some mutations might disrupt its phosphorylation and, thus, its function. Telethonin interacts with the muscle LIM protein (MLP), together with actinin, MLP, Titin, and telethonin might form a complex that senses mechanical stretch^50^.

#### N2-B region

Cardiac-specific N2-B region which made up of Ig-domains can bind to two isoforms of the LIM domain protein, FHL-1 and FHL-2 which respond strongly to biomechanical stress, and can move to the nucleus to work as transcriptional co-activators. FHL-2's activity could suppress calcineurin, inhibiting pathological cardiac growth while FHL1 might connect to the MAPK signaling cascade. Under non-stimulating conditions, MEK1/2 anchors ERK in the cytoplasm, but after activation, it shifts ERK to the nucleus, activating specific transcription factors.

ERK2 has been seen to phosphorylate Titin's N2-Bus sequence, potentially affecting myofilament stiffness. Knocking down FHL1 in mice changed myofibrillar responsiveness and reduced hypertrophic signaling. Hence, the N2-B/FHL-1/MAPK complex might be a key biomechanical stress sensor in cardiomyocytes^44,58,137,189,190^.

#### M-band region

The M-band region of Titin, particularly the Titin kinase (TK) domain, is a significant area for hypertrophic signaling. TK's conformational changes, suggesting its role as a biomechanical stress sensor, might be biomechanically induced. When activated, TK interacts with Nbr1, forming a complex with p62/SQSTM1 and muscle-specific ubiquitin E3 ligases MuRF1, MuRF2, and MuRF3.

The TK signaling complex with the zinc-finger protein nbr1 is involved in mechanically-activated signaling. Nbr1 directs the ubiquitin-binding protein p62/SQSTM1 to sarcomeres where it interacts with the muscle-specific E3 ligase MuRF2, linked to the transactivation domain of serum response factor (SRF). Mechanical inactivity triggers MuRF2 nuclear migration, decreasing nuclear SRF and suppressing transcription. Mutations in the TK domain disrupt this mechanism, resulting in hereditary muscle disorders^50,191^.

Of course, it should be considered that subsequent investigations have proposed that TK functions as an inactive pseudokinase, utilizing its kinase scaffold to recruit MuRF1 for biomechanically regulated autophagy pathways^192,193^.

### The hotspot region for *TTN* variants

In a quantitative analysis of variants, it was revealed that the most common hotspot region for variants is the exon number 326 which is located in the A band as the Fibronectin type III domain^194^ and has a more considerable number of variants compared to other parts which are followed by exon 358 (containing Ig-like domain and Fibronectin type III domain)^194^ and exon 48. Among the introns, intron 47 can be considered as the hotspot point for variants compared to other introns^194^ (Fig. 2).

## Discussion

This study identified 611 distant TTN variants, classified as pathogenic, likely pathogenic, or variants of uncertain significance (VUS). These variants predominantly occurred in exon fragments (85%), with 69.6% classified as pathogenic, 21.6% as likely pathogenic, and 8.8% as VUS in ACMG classification. Substitutions accounted for 57.25% of the variants, deletions for 29.62%, duplications for 7.36%, and insertions for 5.72%. The majority of pathogenic variants were located after exon 326, exhibiting higher CADD scores. GERP scores indicated conservity among gene nucleotides, with most variants having notable GERP scores. Exons at the end of the gene displayed higher average CADD scores. VUS variants had lower CADD scores.

TTN, a functionally and structurally essential component of striated muscles, is the largest human protein^10,11^. It consists of four functional regions including N-terminal, I-band, A-band, and C-terminal^26^. The N-terminal is an anchor for Z-disk, which not only plays a crucial role in myofibril assembly and stability but also in sensory functions, protein interactions, and signaling pathways^32–40^. Owing to alternative splicing, I-band is the central adopter specializing titin for specific tissues. The elasticity of the titin is mostly attributable to the I-band unit^38,41^. On the contrary to the I-band, the A-band is not extensible and is a stable anchor for myosin fibers. It also interacts with various proteins contributing to protein turnover at the sarcomeric center^38,41^. The M-band constitutes the myomesin-titin-myosin and also senses and responds to the metabolic stress^50^.

The passive tension of the human heart is determined by the pattern of expression of titin isoforms. Expression of more elastic and larger I-band isoforms is associated with lower titin passive tension. The ratio of N2BA and N2B isoform expression determines the stiffness of cardiomyocytes^60^. If the balance between N2BA and N2B is disrupted and N2BA isoform upregulates, the decrease in passive stiffness of the heart brings about DCM^30,31,62,63^. Mutations in the TTN gene are speculated to bring about cardiomyopathies through disruption in sarcomere assembly or contractility, or triggering aberrant splicing^30,31,62,63^.

In accordance with our study, another study demonstrated that most TTN variants associated with TTN are located in the A-band unit followed by the I-band^26^. Truncating TTN variants located in the A-band region are the predominant TTN mutations associated with the DCM^77–80,86–88^. The N2BA and N2B isoforms contain distal exons of the A-band. Therefore, variants affecting the A-band and its distal regions are more frequently reported to manifest with DCM, while, the N-terminal mutations are less likely to bring about DCM, considering they are not expressed in N2BA and N2B isoforms^77^.

TTN mutations are not as prominent in HCM compared to DCM. HCM is speculated to arise from mutations in sarcomere-related genes; nonetheless, the exact pathophysiology of HCM is yet to be found^90^. Mutations in Sarcomeric, non-sarcomeric, and sarcomere-associated proteins are proposed to contribute to the development and inheritance of RCM^1,2,106,107^. Although the role of TTN variants in the pathogenesis and inheritance of RCM is not fully understood, it is known that titin is the key determinant of sarcomere resting tension and diastolic function^36,108,109^. Similarly, the impact of TTN mutations in ARVC is not yet determined. However, rare TTN variants have been reported in probands and family members of ARVC patients^121,123^.

The most common hotspot for mutations is exon 326 of the TTN gene which is located in the A-band region. Notably, the exon containing the most TTN variants is 358, also in the A-band. As presented, the TTN variants were primarily located in a small number of exons which are mostly situated at A- and I-bands. This localization of TTN variants might stem from the higher fatality of mutations in other locations, or conversely, these mutations do not exhibit clinical symptoms to prompt genetic evaluation.

The conservatory TTN exons seem to be associated with the pathogenicity of the variants This might be explained, at least in part, by the theory that more conserved nucleotides could be essential, and mutations affecting this nucleotide could be more pathogenic.

## Data Availability

The datasets generated and/or analyzed during the current study are available in the the public archive of interpretations of clinically relevant variants (ClinVar) repository, (https://www.ncbi.nlm.nih.gov/clinvar/?term=TTN%5Bgene%5D&redir=gene).
